# Enhancing primary care for older adults: the safety, efficacy, and adherence (SEA) team-based care model to reduce adverse medication outcomes

**DOI:** 10.3389/fpubh.2025.1453485

**Published:** 2025-08-06

**Authors:** Michael Changaris

**Affiliations:** ^1^Integrated Health Psychology Training Program, The Wright Institute, Berkeley, CA, United States; ^2^Doctor of Psychology Program, University of San Francisco, San Francisco, CA, United States; ^3^John Muir Health, Family Medicine Residency Program, Walnut Creek, CA, United States; ^4^Contra Costa Family Medicine Residency Program, Martinez, CA, United States; ^5^LifeLong Medical Care, Oakland, CA, United States

**Keywords:** gerontology, polypharmacy, geriatric primary care, integrated behavioral health, older adult care, interdisciplinary care, deprescribing, social determinants of health

## Abstract

**Introduction:**

Older adults face significant health risks owing to gaps in the management of polypharmacy and medication adherence, as well as the integration of physical and mental health needs. Current models do not fully address these challenges. This study introduced the Safety, Efficacy, and Adherence (SEA) model designed to enhance interdisciplinary collaboration, improve medication management, and integrate care for older adults. This model addresses the core drivers of poor health outcomes: (1) medication adherence challenges, (2) social determinants of health, (3) polypharmacy, (4) team-based care with family support for deprescribing, and (5) psychosocial factors related to aging.

**Methods:**

The SEA model was developed through a structured literature review focusing on medication safety, polypharmacy, behavioral health integration, home safety inspections and adherence. It draws on frameworks such as the Chronic Care Model, Interprofessional Collaborative Care for Older Adults, and Consolidated Framework for Implementation Research. This model fosters interdisciplinary collaboration by integrating pharmacists, primary care providers, mental health professionals, substance use treatment, and family suppowrt, and it is adaptable to diverse clinical settings.

**Results:**

The SEA model assessed short- and long-term outcomes. Potential short-term effects included improved medication adherence, enhanced team coordination, and reduced occurrence of adverse drug events. Long-term goals and possible effects included better chronic disease management, fewer hospitalizations, and improved quality of life for older adults. The model’s scalability allows for application across various healthcare settings, although further testing is required for validation.

**Conclusion:**

The SEA model provides a comprehensive framework for addressing the complex needs of older adults by focusing on medication SEA. Two vignettes, one clinical and one organizational, demonstrate the practical application of the model in patient care and implementation science. By improving interdisciplinary collaboration and addressing social and behavioral factors, in home safety for medications, this model aims to reduce polypharmacy and hospitalization. Based on existing evidence-based frameworks, this model would benefit from future studies to validate its effectiveness in diverse settings.

## 1 Introduction

Over the past two centuries, global life expectancy has increased dramatically, from approximately 30 to 72 years (1, 2). As a result, more adults over the age of 60 are alive today than ever before. However, this increase in lifespan has not been matched by an equivalent increase in healthspan—the number of years lived in good health (3, 4). This widening gap presents both challenges and opportunities for prevention, care delivery, and health system redesign (5–7).

The SEA model brings systems together to address fragmentation. Many healthcare organizations struggle to meet older adults’ complex needs, leading to preventable illness and hospitalization. Fragmented care, workforce shortages, and training gaps compound these challenges. Team development strategies offer a path forward (8).

As the population continues to age, healthcare systems face rising demands, intensified by a persistent shortage of geriatric specialists and insufficient training in aging-related care among primary care and behavioral health providers (4). Structural inequities—including exposure to racism, sexism, ageism, and other forms of bias—further shape older adults’ access to care, treatment experiences, and long-term outcomes. While services may be available, access alone does not guarantee effective or equitable utilization—especially when care is fragmented or culturally unresponsive (8).

Primary care providers (PCPs)—who serve as the frontline for most older adults—are increasingly expected to manage complex concerns such as polypharmacy, medication safety, and adherence. However, they often do so without adequate support: geriatric-focused training, interdisciplinary coordination, and access to care extension teams such as behavioral health providers and pharmacists remain unevenly distributed (9–13). These systemic gaps contribute to functional decline, preventable hospitalizations, and reduced quality of life for many older adults.

The SEA model is designed to simplify and unify care delivery for older adults. It integrates behavioral, physical, and psychosocial care into a single, coordinated workflow grounded in four core elements: (1) timely communication across team members, (2) structured shared decision-making with patients and families, (3) targeted training to address persistent care quality gaps, and (4) local adaptation through improvement-science strategies. By aligning medication safety efforts with attention to social determinants of health and interdisciplinary collaboration, SEA reduces fragmentation, duplication, and disjointed handoffs. Its structure clarifies roles, enables real-time coordination, and promotes more cohesive, person-centered care.

Addressing these gaps by increasing the number of clinicians with geriatric prescribing expertise is necessary but insufficient. To truly improve care, we must understand the structural and intersectional realities—including racism, sexism, ageism, and other forms of oppression—that shape older adults’ experiences. Through targeted training, practical tools, and equity-focused metrics, the SEA model works to equip primary care teams to deliver culturally responsive, justice-oriented care that improves outcomes across diverse populations (14).

Strategies such as academic detailing, clinical champion engagement, and targeted residency training for family medicine practitioners can improve medication management and support aging patients more holistically. This includes addressing mental health needs, substance use challenges, and the complex dynamics faced by families and caregivers. Together, these efforts enhance quality of life and promote more responsive, integrated care (7, 10, 14).

Academic detailing is a powerful strategy for bringing interdisciplinary teams together to defragment care, coordinate treatment decisions, and improve outcomes for older adults. It offers personalized, face-to-face education to clinicians within team-based care settings, aligning approaches across disciplines (15). In older adult care, academic detailing provides real-time, targeted education. It helps teams adapt treatment as patients’ cognitive, functional, and social needs evolve (5, 15–18). Unlike traditional lecture formats, it emphasizes active learning, enabling clinicians to engage in problem-solving and receive immediate feedback (16).

Interdisciplinary teams trained through academic detailing are better equipped to integrate evidence-based practices (EBPs) into daily workflows, enhancing coordination and clinical precision across roles (17, 19). Studies show that up-to-date teams not only demonstrate greater adherence to clinical guidelines, but also achieve superior outcomes for patients with complex care needs. Academic detailing draws on adult learning theory allowing it to address both attitudinal and structural barriers to clinician behavior change (15). This is especially critical in geriatric care, where effective interventions must respond to a range of interconnected concerns—such as managing multiple medications, addressing cognitive decline and chronic conditions like hypertension or diabetes, and recognizing the intersectional effects of ageism, marginalization, and systemic oppression.

Academic detailing provides targeted, real-time education to interdisciplinary teams and supports the uptake of evidence-based practices into routine care (15–17). This strategy enables teams to integrate clinical updates—such as changes in the Beers Criteria (16, 20)—and tailor treatment decisions to the complex medication management needs of older adults (21–23). It integrates evidence-based team strategies that enhance cohesion, reduce interprofessional conflict, and align diverse roles around shared goals and structured care pathways, ultimately improving coordination and clinical outcomes.

Multiple factors contribute to necessary and unnecessary polypharmacy in older adults, including multimorbidity, age-related physiological changes, involvement of multiple prescribers, guideline-driven care, over-the-counter medication use, mental health challenges, acute episodes, and prescribing cascades triggered by side effects (21, 24). The absence of structured deprescribing and insufficient medication review further increase the risk of harm (5–7, 15, 16, 25). Academic detailing offers a collaborative, interdisciplinary process that brings teams together to tackle these medication-related risks.

Organizations such as the American Medical Association and the World Health Organization have called for structured, continuous education to support team-based, person-centered care (26, 27). The SEA model responds directly to this need by embedding practical tools and equity-focused training into everyday workflows. Drawing on principles from implementation science, SEA helps teams build skills over time through adaptable strategies and feedback, supporting high-quality geriatric care and long-term sustainability in under-resourced settings.

Clinical champions—healthcare professionals who assume leadership roles to promote evidence-based practices (EBPs)—are key to addressing these gaps. Their responsibilities include facilitating development of clinic specific academic detailing, developing site-specific workflows, and using electronic medical record (EMR) tools and flags for medication safety. In older adult care, champions can serve as a bridge between frontline teams and leadership, helping integrate quality improvement efforts and foster clinician engagement. Clinical champions help spread the Safety, Efficacy, and Adherence (SEA) model, tailoring implementation strategies to local needs and system readiness (20, 28, 29).

This study proposes the SEA model, a team-based care framework designed to improve medication safety, efficacy, and adherence for older adults. Given the complexity and evolving nature of older adults’ needs, successful care requires more than the transfer of individual knowledge—it requires the development of interdisciplinary systems capable of adapting treatments to real-world clinical and social challenges (5–7, 25, 30). The SEA model builds on established, evidence-informed strategies with strong support from implementation science for improving team-based care, reducing siloed decision-making, and defragmenting communication across disciplines (15–17, 20, 28, 29).

The SEA model is a scalable, adaptable framework that helps healthcare teams across diverse clinics leverage existing roles—e.g., care managers, nurses, and health educators—while building capacity over time. Its ability to flexibly adjust to varying staffing levels, workflows, and resources—supports implementation even in under-resourced settings, though success still depends on infrastructure like staffing, interpreters, and coordination systems. Grounded in clinical evidence, geriatric guidelines, and implementation science, SEA addresses key challenges in older adult care and provides a foundation for ongoing research and scalable implementation in aging populations.

## 2 Methods

### 2.1 Development of the SEA model: a structured review and framework adaptation

This study developed the SEA model by synthesizing evidence from clinical guidelines, implementation science frameworks, and research studies focused on medication management and interdisciplinary care for older adults (8). The SEA model addresses key challenges, including polypharmacy, medication adherence, and social determinants of health (SDOH), with a focus on improving outcomes for older adults with complex chronic conditions. The methodology was structured around focus areas essential for enhancing geriatric care.

### 2.2 Literature review process

To inform the SEA model architecture, we conducted a structured, non-systematic review of empirical and conceptual literature on medication-related risk factors in older adults. The review centered on 10 predefined domains selected for their relevance to medication safety, implementation barriers, and system-level gaps. Domains were identified through review process and existing implementation frameworks.

**Figure 1:**
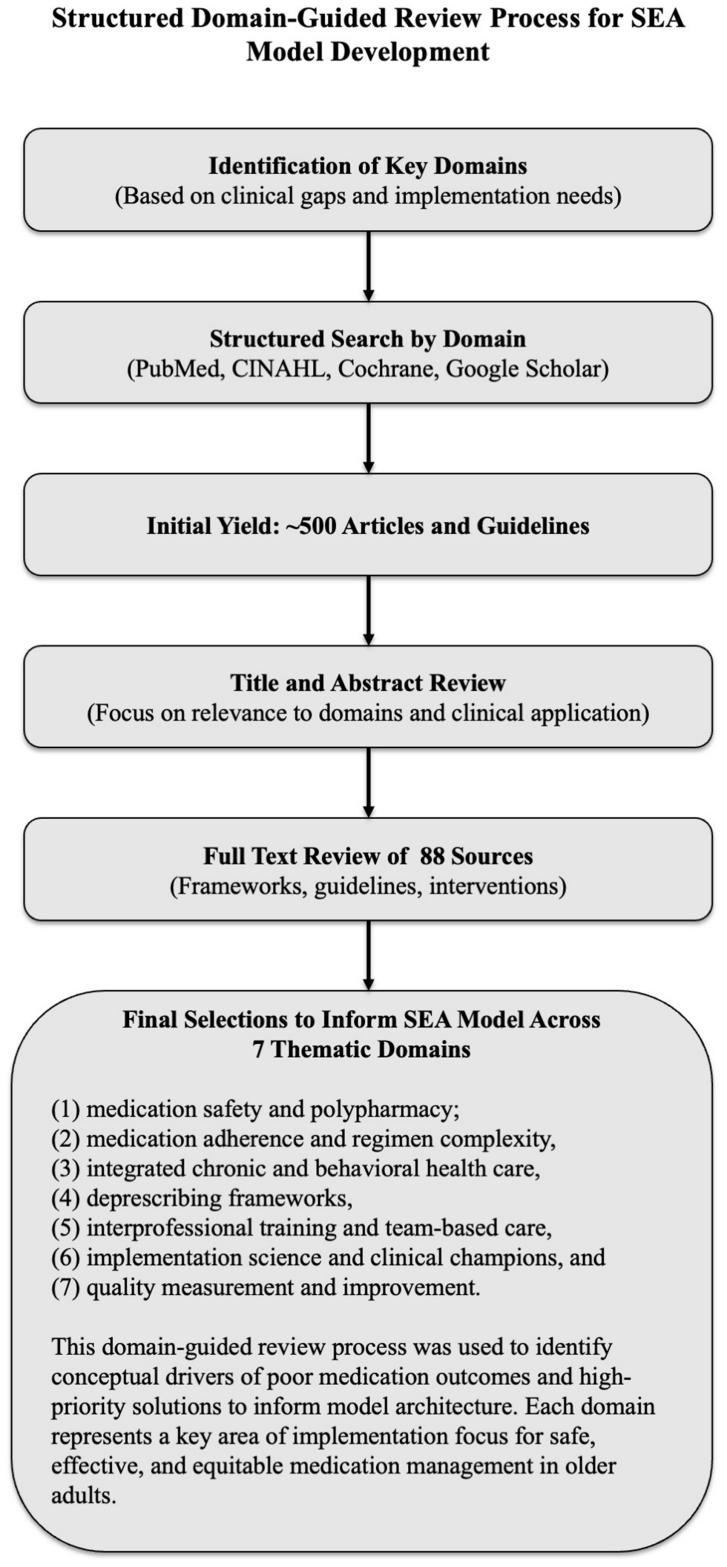
Structured domain-guided literature review process for SEA model development. A structured, non-systematic literature review—using semi-structured searches in PubMed, CINAHL, the Cochrane Library, and Google Scholar (2000–2024)—was organized around seven predefined domains selected for their relevance to implementation gaps in older-adult care: (1) medication safety and polypharmacy, (2) medication adherence and regimen complexity, (3) integrated chronic and behavioral health care, (4) deprescribing frameworks, (5) interprofessional training and team-based care, (6) implementation science and clinical champions, and (7) quality measurement and improvement. Evidence drawn from these domains provided the empirical foundation for the Safety, Efficacy, and Adherence (SEA) model’s conceptual architecture.

The process (see Figure 1) included semi-structured searches of PubMed, CINAHL, the Cochrane Library, and Google Scholar, spanning 2000–2024. Over 500 articles and guidelines were screened. Of those, 88 sources were selected for full-text review based on conceptual clarity, clinical utility, and implementation relevance. The screening and full-text reviews were conducted by the first author, who has clinical and research expertise in integrated behavioral health, geriatrics, and implementation science.

The review adhered to best practices for narrative reviews, including SANRA criteria and EQUATOR Network guidelines. Seven domains were identified *a priori* to guide the process: (1) medication safety and polypharmacy, (2) adherence and regimen complexity, (3) integrated chronic care, (4) behavioral health integration, (5) deprescribing frameworks, (6) interprofessional training, and (7) implementation science. This structured, domain-based approach is depicted in Figure 1.

#### 2.2.1 Medication safety and polypharmacy

This domain emphasized polypharmacy risks and adverse drug events (ADEs). The Beers Criteria offered key evidence-based deprescribing recommendations (18, 31). These needs often extend beyond clinical settings and include supports such as home safety inspections to reduce fall risk, ensure medication access, and prevent avoidable hospitalizations.

#### 2.2.2 Medication adherence

Barriers to medication adherence include regimen complexity, cognitive decline, cultural and structural inequities—including racism, ageism, and economic stress. Trials emphasize education, caregiver engagement, and regimen simplification, though effectiveness varies (11, 22).

#### 2.2.3 Integrated chronic care

Team-based models for chronic disease (e.g., diabetes, hypertension) showed promise but vary in design and generalizability (25, 32–34).

#### 2.2.4 Patient-centered models

Frameworks like the Chronic Care Model and PCMH aligned with SEA’s emphasis on coordinated, patient-driven care (25, 30, 32–34). SEA extends these models integrating age-informed care, equity-focused training, and practical tools for addressing polypharmacy, caregiver dynamics, and social determinants of health in older adults.

#### 2.2.5 Interprofessional training

Findings underscored the need for collaborative skills in managing polypharmacy, cognitive health, and older adult care (14, 35–37).

#### 2.2.6 Deprescribing frameworks

Strategies focused on safe medication reduction amid multimorbidity. Balancing symptom control and polypharmacy remains challenging (10, 38, 39).

#### 2.2.7 Behavioral health integration

Primary care models addressing depression, anxiety, and dementia are promising but require broader scaling and validation (25, 33, 40, 41).

#### 2.2.8 Quality metrics and evaluation

Key indicators (e.g., readmissions, adherence, and satisfaction) informed the SEA model’s outcomes framework (42).

The SEA model reflects a synthesis of this literature and available implementation guidance. Its development is iterative, acknowledging current knowledge limits and the need for continued refinement across diverse healthcare settings.

### 2.3 Framework development

The SEA model development was guided by key principles from existing frameworks such as the Collaborative Care Model (CoCM), Patient-Centered Medical Home (PCMH), and implementation science frameworks like the Consolidated Framework for Implementation Research (CFIR) (32, 34, 43). These models emphasize patient-centered care, culturally responsive practices, interdisciplinary collaboration, and the use of evidence-based practices—all of which align with SEA’s foundation.

However, SEA extends these frameworks by incorporating geriatric-specific priorities—such as cognitive impairment, caregiver strain, home safety and polypharmacy—while also embedding culturally responsive care, equity-focused training, and implementation supports designed to meet the needs of adults from traditionally marginalized communities.

The model also draws on insights from real-world quality improvement projects, particularly those aimed at reducing hospitalizations, improving medication adherence, and strengthening care coordination—core elements that shape SEA’s practical application.

### 2.4 Implementation science approach to SEA: 4Ts framework

A semi-structured scoping review of 60 peer-reviewed studies and four geriatric guidelines revealed four persistent barriers to safe, effective medication use in older adults. First, training in geropharmacology and motivational interviewing remains limited across many care settings (5, 17, 44). Second, care pathways are fragmented—deprescribing, cognitive screening, and social-risk assessment often occur in isolation (10, 18, 38). Third, clinicians rely on quarterly rather than real-time data, weakening point-of-care tracking (42, 45). Finally, fourth accountability for medication management is diffuse, which undermines team coordination and continuity of care (28, 46, 47).

The implementation framework translates the SEA (Safety, Efficacy, and Adherence) model into practice by integrating four key strategies that support clinical decision-making and patient-centered care. First, interdisciplinary teams receive focused training in geriatric pharmacology and motivational interviewing, building the foundation for safe, tailored prescribing (17, 25, 48). Treatment decisions draw on real-time data from structured assessments. Key inputs include Beers Criteria alerts (potentially inappropriate medications), medication adherence screener scores, and cognitive capacity measures, which are reviewed in team-based deprescribing huddles (16, 49, 50). These assessments are embedded into team-based deprescribing huddles to support timely, collaborative decisions.

To track outcomes, electronic medical record (EMR) dashboards are transformed into dynamic run charts that monitor adherence, polypharmacy, and adverse drug events (ADEs), allowing care teams to spot emerging risks and adjust treatment plans accordingly (25, 51). Team roles are clearly defined: if available in system pharmacists lead pre-visit medication reviews; behavioral health clinicians provide coaching and follow-up for adherence; care managers address social determinants of health (SDOH); and nurses conduct medication reconciliation. Because these processes align with quality indicators from the Centers for Medicare and Medicaid Services (CMS) and the Health Resources and Services Administration (HRSA), the framework not only improves care but also meets benchmarks for value-based reimbursement (5, 6, 23, 25, 52).

Given the current political and economic climate, many social services that older adults rely on, such as transportation, housing support, and in-home care, are being reduced or destabilized. As these systems erode, SEA implementation requires care teams to be increasingly inventive and collaborative in addressing unmet needs.

### 2.5 Integration and adaptation

The SEA model was designed to be adaptable across healthcare settings, ranging from primary care clinics to integrated healthcare systems. Case studies of successful integration into diverse clinical environments were reviewed to ensure applicability. These case studies demonstrated how teams address polypharmacy, medication efficacy, and adherence challenges in older adults, informing the design of the SEA model (9, 25, 32–34).

The model was iteratively refined, whereby the core components of the SEA model were tested against case studies and literature to ensure practicality and scalability. Although the SEA model has not yet been directly validated, it is built on existing research-based models for addressing medication safety in older adults. This model was designed to support further empirical testing, enabling validation and further adaptation across healthcare settings.

## 3 Results

### 3.1 Development of the SEA model for improving older-adult care

Management of older adults presents distinct challenges, including higher ADE rates, complex multimorbidity, and limited access to clinicians trained in geriatric care. These factors contribute to poorer health outcomes and increased healthcare utilization. The SEA model offers a structured, three-phase, team-based framework that integrates principles of implementation science with evidence-based geriatric best practices. This approach directly addresses the primary drivers of poor outcomes in later life—polypharmacy, social determinants of health (SDOH)-related barriers, cognitive and mental health comorbidities, and fragmented care coordination (33, 34, 37, 39, 41, 42).

In the safety phase, the model clarifies distinct yet coordinated roles across the care team. Pharmacists lead medication safety reviews, flag high-risk medications, and recommend medication consolidation strategies to minimize ADEs and hospitalizations (18, 37, 39). Physicians integrate these recommendations into clinical decision-making, assess physiological risks (e.g., falls, overdose, capacity concerns, home safety), and adjust prescriptions within the broader context of patient care creating a safety net for medication management.

In the efficacy phase, pharmacists and physicians collaborate to implement age-adjusted dosing and deprescribing strategies that address metabolic, functional, and cognitive changes, supporting the long-term management of chronic conditions (25, 32–34).

During the adherence phase, behavioral health specialists, nursing staff, and health educators help patients navigate regimen complexity, cultural and social barriers, and gaps in support. Behavioral health providers also strengthen motivation and self-management, supporting long-term engagement in care (11, 22, 25, 32–34, 42). Barriers to adherence often include complex regimens, cognitive decline, cultural mismatches, and limited support. These are compounded by systemic inequities—such as racism, ageism, gender bias, and the intersectional effects of economic stress and marginalization. The People–Access–Commitment–Systems (PACS) mnemonic offers four key targets for teams to assess and address: People (social supports), Access (SDOH-related barriers), Commitment (motivation and beliefs), and Systems (tools to manage complex regimens).

**Figure 2:**
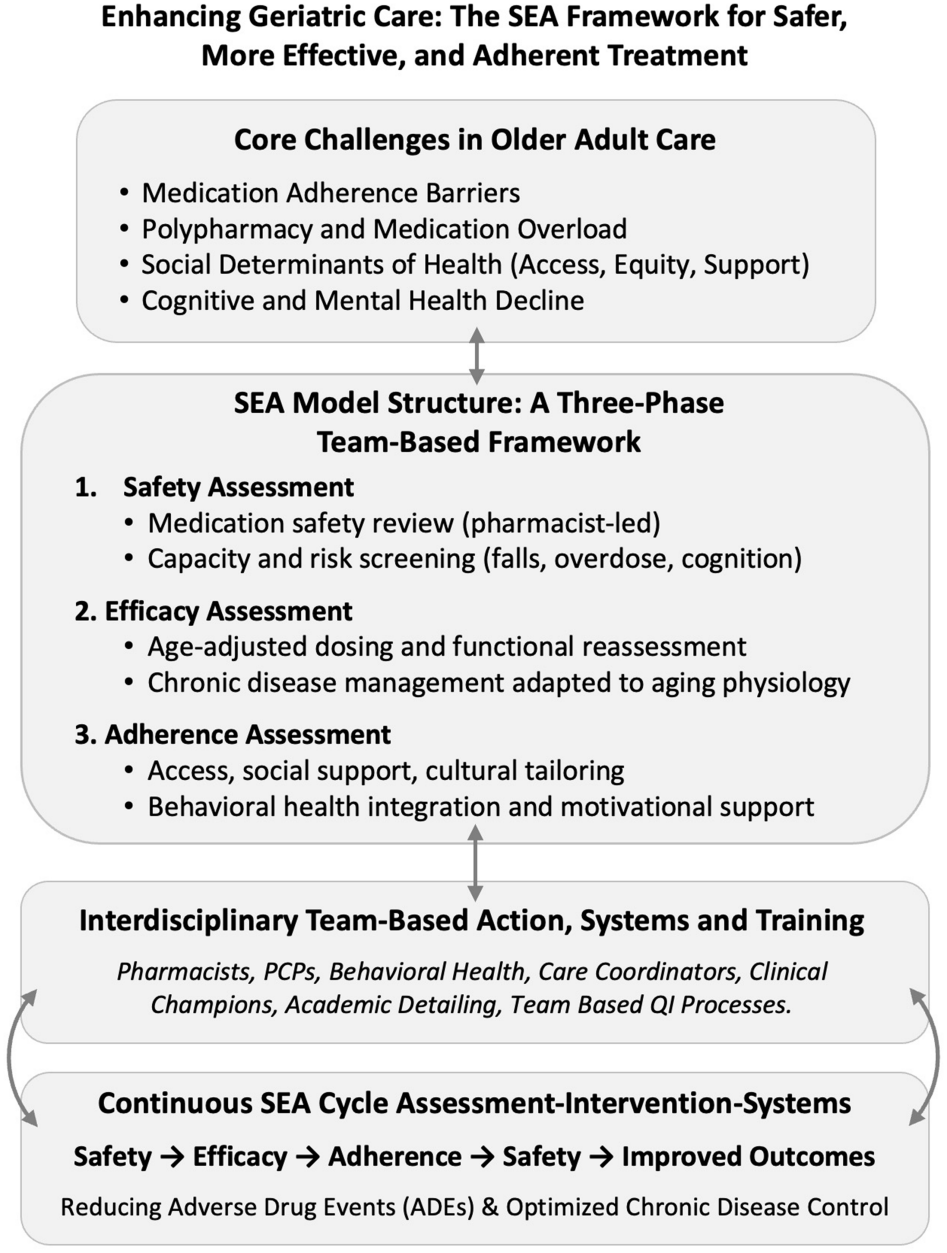
Enhancing geriatric care the SEA framework for safer more effective and adherent treatment. Enhancing geriatric care: the SEA framework for safer, more effective, and adherent treatment. Structured around these pillars, SEA lowers ADE rates, improves disease control, raises patient satisfaction, and delivers measurable quality-metric gains. Clinical champions and academic-detailing sessions translate annual updates—e.g., the 2023 Beers Criteria and STOPP/START tools—into daily workflows, sustaining momentum across PCPs, pharmacists, behavioral-health clinicians, and care managers (25, 28, 33, 37, 41). ADE, adverse drug event; PCP, primary care provider; SEA, Safety, Efficacy, and Adherence; STOPP/START, Screening Tool of Older Persons’ Prescriptions/Screening Tool to Alert to Right Treatment.

The SEA model also supports the development of Electronic Medical Record (EMR) tools that automate safety alerts and Clinical Decision Support (CDS). These tools flag high-risk medications, fall and overdose risks, and capacity concerns while identifying through SDOH, limited social support, and prior nonadherence indicators patients vulnerable to poor adherence. Embedded decision supports enable real-time risk stratification and timely SEA-based interventions, offering a structured, team-based pathway to reduce adverse outcomes and improve function and quality of life (Figure 2).

Deprescribing is often complex, requiring the navigation of clinical challenges; communication failures and lack of team coordination remain core drivers of medical errors in the United States, as first highlighted in the *Institute of Medicine’s* seminal report *To Err is Human* (10, 42, 43). Team-based approaches consistently demonstrate better outcomes and improved safety, particularly when addressing high-risk prescribing. The SEA framework supports collaborative medication management and deprescribing. Its “no wrong door” safety approach allows any team member to trigger reviews, helping identify risks and opportunities for improvement (42, 43).

In practice, when any clinician flags a Beers-listed medication, duplicate therapy, or new ADEs during the safety phase, a rapid deprescribing huddle is convened. This may occur through EMR-based team messaging or, when complexity warrants, in-person meetings for collaborative consultation. While clinics are supported to adapt processes to local resources, SEA outlines key roles: pharmacists review risks, physicians align changes with goals, behavioral health clinicians address taper-related anxiety, and care managers coordinate follow-up and stop auto-refills (10, 18, 25, 33). This structured approach ensures medication changes occur within a continuous, team-based evaluation cycle (39, 42).

Interprofessional collaboration—among pharmacists, primary care providers, and mental health clinicians—is central to SEA and strengthens care coordination, improving adherence, satisfaction, and reducing hospitalizations (25, 33, 38, 41–43). The following section presents findings from the structured review that informed the model’s development, identifying key drivers of harm and ways to reduce care fragmentation.

### 3.2 Drivers of medication risk and system breakdown in older adults: insights from structured review

During development of the SEA (Safety, Efficacy, and Adherence) model, a structured scoping review of 88 peer-reviewed studies was conducted, national clinical guidelines, and interdisciplinary care initiatives to identify the most frequently cited drivers of poor medication outcomes in older adults (see Section 2.2). This process aimed to ensure the model was both evidence-based and responsive to the realities of team-based clinical practice.

To organize the model and enhance its relevance, SEA domains were aligned with three foundational frameworks in geriatric and implementation science. The Chronic Care Model (CCM) emphasizes proactive, team-based management of complex chronic conditions like polypharmacy (34). The Consolidated Framework for Implementation Research (CFIR) offers a lens for translating evidence into practice (28). The Interprofessional Curriculum for the Care of Older Adults (iCCOA) outlines core competencies for coordinated, high-quality care (35, 37). Building on these, SEA extends their principles by embedding structured training, defined roles, and real-time decision-making into workflows aligned with value-based care. Supplementary Tables 1, 2 map these alignments.

Medication adherence is a frequent and consequential challenge. Fewer than half of older adults follow prescribed regimens, contributing to 10% of hospitalizations (11, 18). Common causes include cognitive decline, complex regimens, and low health literacy (13, 22, 37). SEA’s Adherence domain addresses these through behavioral strategies and culturally responsive team planning.

Housing insecurity, home safety, transportation gaps, food insecurity, and financial constraints—were consistently linked to medication access, treatment interruptions, and lower-quality prescribing (53–56). These factors disproportionately affect marginalized older adults and amplify the risks associated with polypharmacy and care fragmentation. SEA’s Safety and Efficacy domains incorporate these social determinants of health (SDOH) into screening, home safety assessments and support planning to address these gaps (12).

Polypharmacy, defined as the concurrent use of five or more medications, affects an estimated 44% of older adults; over 20% meet hyperpolypharmacy criteria (57). The risks of drug–drug interactions, ADEs, and functional decline increase substantially with each additional medication. Literature reviews consistently point to team-based medication reviews, deprescribing protocols, and pharmacist involvement as critical mitigators and are central to SEA’s Safety component (10, 39).

Deprescribing is a complex, high-stakes process. Though it reduces adverse drug events (ADEs), it can also trigger withdrawal, symptom return, or anxiety—especially without structured follow-up (23). SEA embeds deprescribing within an interdisciplinary model, using defined roles and behavioral health integration to ensure safety and effectiveness (10, 12).

Psychosocial and mental health factors—including trauma, depression, social isolation, and lack of family engagement—interfere with medication adherence, care engagement, and decision-making (13, 52, 58, 59). Whereas these factors are frequently underdiagnosed or deprioritized in standard geriatric care models, SEA’s Adherence pillar addresses them by embedding behavioral health clinicians, social supports, and culturally responsive engagement strategies within the team structure.

These domains capture key contributors to preventable harm in older adult care. Their interdependence highlights the need for a structured, scalable model. SEA meets this need by translating evidence into a continuous cycle of team-driven improvement.

### 3.3 Operationalizing the SEA model through teamwork, triage, and adaptive infrastructure

To address these outlined core risk domains, the SEA model provides an integrated, team-based framework for translating evidence into real-world practice. Grounded in structured training, coordinated clinical roles, and adaptive workflows, this model is not just a conceptual model—it is a scalable infrastructure for improving outcomes across primary and integrated care settings. This section outlines how SEA is operationalized, with each domain—Safety, Efficacy, and Adherence—mapped to specific tools, professional roles, and measurable outcomes.

#### 3.3.1 Safety: managing risk through team-based interventions

Older adults face heightened vulnerability to ADEs due to age-related physiological changes affecting drug metabolism, excretion, and central nervous system sensitivity (28, 39, 45). This vulnerability is further compounded by polypharmacy, cognitive decline, and fragmented care systems (10, 13). The SEA model addresses these risks through coordinated, role-specific interventions that operationalize safety as an active, continuous clinical process embedded within routine care.

Pharmacists play a central role by leading structured medication safety reviews, applying validated tools such as the Beers Criteria, START/STOPP protocols (Screening Tool of Older Persons’ potentially inappropriate Prescriptions/Screening Tool to Alert doctors to the Right Treatment), and the Medication Appropriateness Index to systematically screen for high-risk prescribing patterns (18, 37, 39). These reviews are supported by clinical tools including the Naranjo Adverse Drug Reaction Probability Scale, which assesses the likelihood of drug-related harms, and age-specific frailty indices that contextualize medication risks within the broader health status of older adults (51). Many clinics lack structured processes that fully integrate pharmacists or medication review into care management, resulting in missed opportunities to reduce polypharmacy and medication-related harm.

Team-based processes—such as medication safety huddles, structured chart reviews, and shared communication workflows—are central to how SEA manages deprescribing and medication safety. Chart reviews use validated tools to flag high-risk medications. Communication workflows, including EMR alerts, care plans, and warm handoffs, support real-time team coordination. Risk flags trigger interventions like pharmacist–PCP coordination, patient and family engagement, or interdisciplinary deprescribing huddles.

Deprescribing huddles are brief, focused team meetings—synchronous or asynchronous via EMR—that address medication-related risks. Often pharmacists lead with risk–benefit analyses and deprescribing plans; primary care providers align these with patient goals through shared decision-making. Nurses monitor for sedation, confusion, or fall risk, triggering safety reviews. Implementation science strategies embed these practices into daily workflows, improving communication, team cohesion, and collaborative care planning.

Integrated Behavioral Health (IBH) clinicians contribute by preparing patients and families for the psychological impacts of medication changes, providing anticipatory guidance around tapering anxiety and symptom re-emergence. Utilizing evidence-based approaches such as motivational interviewing and brief cognitive-behavioral interventions, IBH providers address emotional barriers that often undermine prescription adherence (41, 48). Health educators can reinforce these processes by delivering personalized medication literacy counseling, facilitated medical group visits. Developing patient-centered medication schedules that fit daily routines, and providing culturally responsive education to address beliefs and misconceptions about pharmacotherapy (19).

These team-based safety efforts rely on adaptive clinical infrastructure supported by EMR functionality. Clinical decision support flags medication risks (18, 42). Integrated huddle boards, structured messaging (42, 60), and standardized templates (e.g., “Deprescribing Consult Note”) ensure SEA interventions are documented, communicated, and evaluated consistently.

Through this integrated proactive approach, the Safety, Efficacy, and Adherence (SEA) model transforms medication safety from a retrospective analysis into a dynamic, team-driven process. This model reduces pill burden, prevents prescribing cascades, and supports functional preservation and improves Quality of Life (QOL) for older adults (39, 42).

#### 3.3.2 Efficacy: adapting care to aging physiology and functional needs

Effective treatment for older adults requires continuous adaptation to evolving biological, functional, and psychosocial realities (21, 61). Medications may lose efficacy or introduce new risks as disease states progress, organ systems decline, and multimorbidity emerges (20, 33). While the SEA model offers a structured framework, clinical judgment must tailor efficacy evaluations to individual patient contexts to ensure relevance and appropriateness (21, 61). The model embeds efficacy evaluations directly into clinical workflows aligning interventions with both physiological changes and patient-centered goals (12, 62).

Pharmacists and physicians lead efficacy reviews and can build context-specific toolkits using SEA’s available assessments, including the Anticholinergic Burden Index, Clinical Frailty Scale, and Functional Comorbidity Index to evaluate risk and treatment burden (61). Deprescribing strategies, guided by the Deprescribing Risk Evaluation Checklist, help reduce unnecessary medications or transition to safer options (12, 17). EMR-integrated clinical decision support flags high-risk drugs and prompts review as conditions change (23, 62).

Efficacy is further monitored through age-adjusted clinical targets—e.g., relaxed systolic blood pressure goals, individualized hemoglobin A1c thresholds, and de-escalation of lipid management intensity—reflecting the latest geriatric consensus guidelines (18). Functional status assessments (e.g., gait speed, activities of daily living [ADL]/integrated ADL scales), cognitive screening (MoCA, Mini-Cog), and symptom tracking determine the real-world effects of pharmacologic and non-pharmacologic interventions (6, 7).

In this model, physicians guide therapeutic decision-making through structured shared-decision frameworks for interdisciplinary teams that keep treatment intensity aligned with life expectancy, functional goals, and patient preferences (25). Two well-validated tools illustrate this approach. The Ottawa Decision Support Framework (63) offers practical criteria for clarifying decisional conflict and tailoring support. The SHARE Approach—an evidence-based, five-step method from the Agency for Healthcare Research and Quality (AHRQ)—guides clinicians to seek patient participation, help explore options, assess values and preferences, reach a joint decision, and evaluate that decision (64). Embedded within the SEA model, these frameworks enable interdisciplinary teams to balance guideline-based prescribing with the lived experiences and treatment goals of each patient, strengthening the person-centered core of medication management.

Through case reviews and EMR alerts, SEA teams help older adults avoid overtreatment and stay aligned with current evidence. This ongoing efficacy cycle counters therapeutic inertia, improving healthcare use, function, and quality of life (6, 12).

#### 3.3.3 Adherence: supporting access, systems, and motivation

Adherence remains a cornerstone of effective care, yet only 45% of older adults maintain high adherence to prescribed regimens, and nonadherence contributes to nearly 10% of hospitalizations in this population (11, 22). The SEA model reframes non-adherence not as a patient failing but as a systemic care challenge shaped by cognitive capacity, health literacy, cultural beliefs, and social context (20). This paradigm shift enables teams to move beyond compliance-focused interventions and implement structural, behavioral, and motivational strategies to support sustainable engagement.

Within the Safety, Efficacy, and Adherence (SEA) model, adherence and cognitive assessments are embedded into team workflows to keep medication plans in line with each patient’s capacity and support needs. These assessments identify behavioral, psychological, and cognitive barriers, allowing real-time treatment adjustments that protect patient safety.

To carry out these assessments, the SEA model employs validated adherence tools tailored to older adult populations. Core adherence tools include the 8-item Morisky Medication Adherence Scale (MMAS-8), reliable across diverse older-adult populations (49), and the Beliefs about Medicines Questionnaire (BMQ), which captures patients’ beliefs about their medicines (65). When administered by behavioral-health clinicians, nurses, or pharmacists, these instruments uncover issues such as forgotten doses, medication mistrust, or side-effect fears.

In team-based settings such as integrated behavioral health (IBH), trained professionals—behavioral-health clinicians, pharmacists, or nurses—reduce physician burden by administering and scoring these tools. Results are entered into the shared electronic medical record (EMR), where clinical-decision-support (CDS) alerts flag low-adherence scores and can prompt a regimen review or motivational-interviewing referral. This closed loop improves communication, workflow integration, and timely treatment adjustment.

Cognitive screening ensures medication plans match a patient’s functional capacity. Brief tools such as the Mini-Cog (three-item recall plus clock drawing) (66) and the Montreal Cognitive Assessment (MoCA) (50) detect early cognitive decline; when impairment is identified, the team may simplify regimens, synchronize refills, or involve caregivers for daily support. All assessments can be adapted for language, culture, and literacy, ensuring equitable, patient-centered care across diverse populations.

SEA teams implement multifaceted, interdisciplinary interventions. Pharmacists deprescribe, consolidate doses, and offer adherence tools like pill organizers and refill syncing (12, 23). Physicians guide shared decision-making to align treatment with patient goals (25, 67). Behavioral health providers use motivational interviewing and cognitive-behavioral strategies to address ambivalence and anxiety, applying models like the Health Belief Model to assess readiness for change (52, 59, 64, 68).

Social workers and care managers assess SDOH and relational supports using tools like the Accountable Health Communities (AHC) Health-Related Social Needs Screening Tool, which evaluates factors such as housing instability, food insecurity, and transportation barriers (69). The Lubben Social Network Scale is also used to assess the size and quality of a patient’s social support network, identifying risks related to isolation and lack of caregiver support (70).

Effective adherence strategies also benefit from robust clinical infrastructure to facilitate communication, monitor risks, and address Social Determinants of Health (SDOH) barriers (56). Advanced EMR systems support these processes through team-based dashboards—e.g., Epic Healthy Planet and Cerner HealtheIntent—providing centralized views of adherence metrics, pending follow-ups, and care coordination activities (23, 62). EMR-integrated secure messaging platforms and task management tools facilitates real-time collaboration among team members helping ensure timely interventions (62, 71). Clinical decision support (CDS) tools and risk-flagging systems help teams identify patients at high risk for medication non-adherence. These algorithms track refill gaps, medication-possession ratios, and missed visits, triggering alerts via EMR refill gap reports and adherence dashboards that notify clinicians of delays or skipped refills (23, 62).

Structured screening for social determinants of health (SDOH)—such as housing, transportation, and financial strain—is now embedded in most electronic medical records (EMRs). Tools like PRAPARE (Protocol for Responding to and Assessing Patients’ Assets, Risks, and Experiences) capture these risks in standardized formats (56, 72). Identified needs can be addressed through referral platforms like Unite Us or FindHelp.org. The Consolidated Framework for Implementation Research (CFIR) helps integrate these tools into workflows, turning SDOH screening and referrals into sustainable, team-based practices.

Integrated care initiatives often falter without structured guidance and change management support. The SEA model (Safety, Efficacy, and Adherence) fills this gap with a stepwise, evidence-based blueprint for embedding medication-management workflows. By aligning training, decision tools, tracking, and team roles within routine care, SEA reduces fragmentation and ensures adherence is supported through real-time communication, actionable data, and proactive social care integration.

#### 3.3.4 Team roles and integrated measurement

The SEA model operationalizes implementation through clearly defined, coordinated clinical roles that support seamless care transitions and continuous monitoring of treatment outcomes (12, 25). Physicians align pharmacotherapy with individualized goals, while pharmacists lead medication safety reviews, efficacy tracking, and interdisciplinary deprescribing huddles (12, 23). Nurses systematically monitor for adverse effects such as falls, fatigue, and cognitive decline. Behavioral health clinicians address adherence barriers using trauma-informed strategies and psychosocial assessments (52). Care managers resolve social determinants of health (SDOH) barriers, coordinate follow-up, and facilitate family engagement (56).

These efforts are supported by structured outcome measurement, including validated tools to monitor cognitive and functional status (e.g., Mini-Cog (66), MoCA (50)) and alignment with age-adjusted clinical targets for A1c, blood pressure, and symptom control (25, 65). Population health management platforms, such as Epic Healthy Planet and Cerner HealtheIntent, integrate clinical decision support (CDS) to flag polypharmacy risks, adherence lapses, and unresolved SDOH factors in real time (23, 56, 62, 73, 74). These alerts enable rapid, team-based intervention to prevent adverse outcomes.

These outcome indicators support internal quality improvement and external reporting, anchoring broader implementation strategies (23, 62). Clinical champions and academic detailing help translate metrics into targeted training and workflow changes (19, 75). By addressing the drivers of harm (Section 3.2) and embedding interdisciplinary feedback loops, SEA promotes safer, more personalized care (12, 23, 25). Its integrated components move the model from theory to practice, offering role-based interventions, validated tools, and scalable performance metrics adaptable to both resource-rich and safety-net settings—supporting equity-focused implementation and ongoing outcomes research (12, 25, 56, 62, 71).

#### 3.3.5 Key performance indicator alignment

The SEA (Safety, Efficacy, and Adherence) model aligns its outcome metrics with several national quality-of-care benchmarks. These include the Centers for Medicare & Medicaid Services (CMS) Star Ratings (76), the Healthcare Effectiveness Data and Information Set (HEDIS) measures (77), and standards from the National Committee for Quality Assurance (NCQA) (78). SEA’s monitoring of high-risk prescribing feeds directly into CMS Star Ratings for medication safety, while its adherence indicators match HEDIS measures for chronic-condition management.

Measures of functional status and cognitive maintenance correspond to NCQA’s person-centered outcome standards—particularly those addressing health-related quality of life (QOL) and independence in older adults (78). The model’s systematic screening of social determinants of health (SDOH) further supports emerging Accountable Care Organization metrics and new CMS requirements aimed at narrowing health-equity gaps (79). By embedding these nationally recognized indicators into everyday workflows, SEA helps health systems improve care quality and meet value-based payment and reporting obligations (23, 34, 62, 71).

Section 3.4 details initial implementation outcomes and outlines continuous-learning pathways. This evaluation strategy uses the Consolidated Framework for Implementation Research (CFIR) and rapid Plan–Do–Study–Act (PDSA) cycles to ensure iterative feedback and sustainable system-level improvement (80, 81).

### 3.4 Translating the SEA model into clinical workflows

The SEA model’s implementation hinges on translating its core principles—safety, efficacy, and adherence—into structured, repeatable workflows within busy primary care settings. This process requires more than education; it demands the intentional redesign of care routines to integrate the complex needs of older adults into daily practice through implementation science strategies, adaptive workflows, and systems thinking (81, 82). Leveraging advanced EMR systems like Epic Healthy Planet (73) and Cerner HealtheIntent (74), along with embedded CDS tools, SEA enables real-time risk detection, proactive medication management, and efficient team communication via structured protocols and integrated messaging (83). When effectively implemented, the SEA model has the potential to extend healthspan, and improve clinical outcomes for community-dwelling older adults.

#### 3.4.1 Operationalizing the model with quality improvement tools

Clinical teams can adopt existing quality improvement structures tailored to aging populations. The PDSA cycle offers a familiar foundation (80, 84). During the Plan phase, teams use screening tools such as the Medication Regimen Complexity Index (85), Adherence to Refills and Medications Scale (49), and Beers Criteria (18) to identify high-risk patients. In the Do phase, interventions are deployed: a pharmacist adjusts a risky regimen; behavioral health clinician applies motivational interviewing (52); and care manager coordinates transportation or conducts home medication inventory. Outcomes—improved adherence, medication reduction, or fewer ADEs—are tracked (Study), allowing iterative refinement (Act) (80, 84).

SEA implementation is strengthened through the presence of clinical champions, typically a physician, pharmacist, nurse, or behavioral health lead who models SEA principles and supports small cycles of change. These champions guide local adaptations, mentor peers, and align the model with clinic realities, increasing buy-in and ensuring contextual fit (81).

#### 3.4.2 Targeting key drivers with driver diagrams

Clinical decision support (CDS) systems help care teams spot patients who are likely to miss doses or stop medications altogether. These algorithms track refill gaps, medication-possession ratios, and missed follow-up visits, then generate alerts for timely outreach (23, 62). For example, the Epic electronic medical record (EMR) platform offers Refill Gap Reports and adherence dashboards that automatically notify clinicians when a patient delays or skips refills.

Structured screening for social determinants of health (SDOH)—conditions such as housing instability, transportation barriers, or financial strain—is now built into many EMRs. A widely adopted instrument, PRAPARE (Protocol for Responding to and Assessing Patients’ Assets, Risks, and Experiences), lets teams record these risks in a standardized format (56, 72). Once needs are identified, referral networks such as Unite Us and FindHelp.org (formerly Aunt Bertha) link patients to food, housing, transport, and financial resources.

Integrated-care studies show that applying implementation-science frameworks—such as the Consolidated Framework for Implementation Research (CFIR) and Plan–Do–Study–Act (PDSA) cycles—improves team coordination and reduces system fragmentation. These principles are built into the SEA model (Safety, Efficacy, and Adherence) to create coherent, team-based geriatric care.

By embedding clinical alerts, social needs screening, and community referrals into structured team workflows, the SEA model transforms disconnected tasks into integrated care processes. This coordinated approach improves medication safety, treatment effectiveness, and sustained adherence in older adults.

#### 3.4.3 Leveraging technology for medication tracking

Technology plays an important role in the SEA model by supporting accurate and timely medication tracking in both clinical settings and home environments. In primary care, tools such as electronic medication administration records, refill-alert systems, and EHR-integrated dashboards help care teams identify potential risks and intervene before adverse medication events occur (9). These systems promote proactive monitoring, allowing clinicians to address adherence challenges early.

At home, digital tools designed for patients and caregivers can extend SEA-based care by simplifying medication routines and reducing cognitive and organizational burden. For example, automated pill dispensers and reminder systems can issue scheduled alerts, monitor dosing behavior, and enable remote monitoring by clinical teams. Simplified packaging, such as pre-sorted, date- and time-stamped packets, may also support more accurate self-administration. Mobile health applications are increasingly used to support communication and coordination among patients, caregivers, and healthcare teams early studies suggest that such digital tools, integrated into work flows can improve adherence (86).

Several implementation challenges persist. Digital literacy gaps, limited internet access, and lack of devices remain common barriers—particularly for older adults and low-income populations (63). Offering multilingual interfaces and culturally responsive design features can improve usability and engagement across diverse communities (87).

Successful adoption also hinges on workflow factors. Reducing alert fatigue, matching each technology to specific team roles, and maintaining staff engagement are critical for sustained use (88). SEA centers an equity-focused roll-out address affordability, infrastructure disparities, and the availability of caregiver support, especially in under-resourced regions. These considerations should guide tool selection and implementation to keep SEA-aligned interventions accessible, acceptable, and effective across patient populations (89).

As the digital health ecosystem continues to evolve, care teams must assess new tools for usability, data privacy compliance, and interoperability with existing clinical systems. Platforms that adhere to interoperability standards—such as Health Level Seven’s Fast Healthcare Interoperability Resources (HL7 FHIR)—offer particular value by supporting seamless data exchange and integration into SEA (Safety, Efficacy, and Adherence) model workflows (60). Regular reassessment of emerging technologies is essential to ensure alignment with the changing needs of older adults and interprofessional care teams.

#### 3.4.4 Involving families in adherence and safety

Older adults frequently depend on informal caregivers—family, friends, or neighbors—for help with medication routines. SEA explicitly integrates caregiver engagement into its Safety and Adherence phases. Clinicians conduct brief caregiver assessments, ensure capacity and clarity in medication plans, and invite caregivers into taper discussions or medication education sessions (26). Pharmacists provide clear medication instructions designed for shared use by patients and caregivers (18). Educational tools, medication calendars, and care-team phone follow-ups support medication-related decision-making. By involving families, teams increase treatment adherence, reduce errors, and strengthen relational support (25).

#### 3.4.5 Sustaining change through champions and detailing

Long-term adoption of SEA depends on building an implementation culture. Clinical champions drive sustained change by modeling SEA-aligned practice, leading huddles, and troubleshooting barriers (20, 28). Academic detailing reinforces this culture with recurring, role-specific updates that translate evidence into action (15, 16). These strategies foster a feedback-rich environment that integrates the SEA model into existing learning, accountability, and care processes (Table 1).

**Table 1:** SEA metrics and screeners to assess risk and monitor outcomes.

SEA pillar	Key risk domains addressed	Screening/assessment tools	Primary team roles	Outcome metrics
Safety	Polypharmacy, inappropriate prescribing, ADE risk, fall risk, cognitive overload	Beers Criteria (18), STOPP/START (51), Naranjo Algorithm (96), Medication Appropriateness Index (97), ARMOR (98), Clinical Frailty Scale (99)	Pharmacists, PCPs, Nurses	ADE rate, # Beers-listed meds, fall incidence, EMR deprescribing flags
Efficacy	Disease control misalignment, functional decline, pharmacodynamic change	Anticholinergic Burden Index (100), DREC (101), Functional Comorbidity Index (102)	PCPs, Pharmacists, Nurses	A1c/BP goals, chronic symptom control, deprescribing success
Adherence	Nonadherence, health literacy barriers, SDOH barriers, social isolation, cognitive impairment	MMAS-8 (61), BARS (85), Beliefs About Medicines Questionnaire (65), Lubben Social Network Scale (70), MoCA (50)/Mini-Cog (66), AHC HRSN Screener (69), PRAPARE (72)	Behavioral Health Providers, Care Managers, Social Workers, Nursing Teams	Adherence rate, refill % data, caregiver-reported concordance, EMR adherence flags

A1c, glycated hemoglobin; ADE, adverse drug event; AHC HRSN, Accountable Health Communities Health-Related Social Needs Screening; ARMOR, Assess, Review, Minimize, Optimize, Reassess (geriatric polypharmacy tool); BARS, Brief Adherence Rating Scale; BP, blood pressure; DREC, Deprescribing Risk Evaluation Checklist; EMR, electronic medical record; MMAS-8, Morisky Medication Adherence Scale (8-item version); MoCA, Montreal Cognitive Assessment; PCP, primary care provider; PRAPARE, Protocol for Responding to and Assessing Patients’ Assets, Risks, and Experiences; SDOH, social determinants of health; SEA, Safety, Efficacy, and Adherence; STOPP/START, Screening Tool of Older Persons’ Prescriptions/Screening Tool to Alert to Right Treatment.

Together, these SEA-informed workflow changes streamline clinical routines and lay the foundation for population-level improvement. By reducing exposure to high-risk medications, enhancing adherence support, and embedding equity-sensitive decision-making, SEA positions primary care systems to lower ADE rates—especially among historically underserved older adults (18, 22, 25, 55). These broader impacts are examined in detail in Section 4.4.

#### 3.4.6 Curriculum and training framework for SEA implementation

Successful SEA implementation likely depends on structured interdisciplinary education and sustained professional development to support competencies in medication safety, care coordination, and patient-centered practices (15, 16). While further research is needed current curricula emphasize the application of validated clinical tools, the development of strong communication skills, and attention to SDOH (25, 26). Additionally, fostering leadership capacity through academic detailing and implementation science principles has been proposed as a means of supporting long-term adoption (20, 28). Table 2 summarizes core training components and key resources intended to guide these efforts.

**Table 2:** Interdisciplinary training components for SEA implementation.

Training focus	Content areas	Key tools and frameworks
Medication safety	Safe prescribing, deprescribing, polypharmacy	Beers Criteria (18), STOPP/START (51)
Care coordination	Structured communication, team collaboration	SBAR (83), TeamSTEPPS (64)
Health literacy and cultural responsiveness	Addressing adherence barriers, patient counseling	Stormacq et al. (75)
Social determinants of health	Screening and intervention strategies	PRAPARE (72), AHC Screening Tool (69)
Behavioral health integration	Motivational interviewing, trauma-informed care	Motivational Interviewing (57)
Cognitive and functional assessment	Screening for cognitive impairment, caregiver engagement	MoCA (50), Mini-Cog (66)
Shared decision-making	Patient-centered treatment planning	Ottawa Decision Support Framework (63), SHARE Approach (64)
Leadership development	Quality improvement, EMR utilization	CFIR (81), PDSA (80), Epic Healthy Planet (73), Cerner HealtheIntent (74)

AHC, Accountable Health Communities; CFIR, Consolidated Framework for Implementation Research; EMR, electronic medical record; MoCA, Montreal Cognitive Assessment; PDSA, Plan–Do–Study–Act; PRAPARE, Protocol for Responding to and Assessing Patients’ Assets, Risks, and Experiences; SBAR, Situation, Background, Assessment, Recommendation; STOPP/START, Screening Tool of Older Persons’ Prescriptions/Screening Tool to Alert to Right Treatment; TeamSTEPPS, Team Strategies and Tools to Enhance Performance and Patient Safety.

The integration of these interdisciplinary competencies not only enhances individual clinical encounters but also strengthens the collective capacity of health systems to reduce adverse medication outcomes at scale.

#### 3.4.7 Scaling SEA for population health impact and health equity

The SEA model moves beyond improving isolated clinical encounters to supporting broader efforts to improve health outcomes at the population level. ADEs represent a leading cause of preventable hospitalizations among U. S. adults aged ≥65 years (9, 18), with the greatest burden consistently falling on low-income and historically marginalized communities (55, 56). This persistent challenge highlights the need for scalable, equity-focused interventions that address both clinical risks and the structural drivers of health disparities.

The following section positions SEA as both a clinical innovation and a potential public health strategy, aligning daily care practices with long-term goals of reducing medication-related harm and advancing health equity (27).

## 4 Discussion: advancing SEA implementation for safer, more equitable medication management

The SEA model (Safety, Efficacy, and Adherence) bridges clinical quality-improvement and public-health equity by using a logic model designed for real-world primary-care settings. It relies on resources most clinics already possess—pharmacist expertise, medication dashboards such as the OCHIN Quality Safety Dashboard, integrated behavioral-health clinicians, and family caregivers—to drive three coordinated care processes (Safety, Efficacy, and Adherence).

These processes yield *actionable outputs*: for example, the number of deprescribing huddles held each month and the change in high-risk prescribing patterns captured by electronic medical-record (EMR) dashboards. Because these indicators are embedded in routine workflows, teams can monitor progress without extra data-collection burden, even in safety-net clinics (81, 83).

Early outputs translate into short-term outcomes—fewer documented medication risks and measurable gains in patient adherence (48, 49). Iterative Efficacy and Adherence cycles, tracked over 3–12 months, are expected to improve chronic-disease control, cut adverse-drug-event (ADE) emergency visits, and boost patient understanding of their regimens (55). Long-term outcomes (> 12 months) include sustained reductions in polypharmacy, lower medication-related readmissions, and better health-related quality of life (QOL) (27). This continuous-feedback approach aligns with implementation-science frameworks (80). Table 3 cross-walks each outcome to the metrics captured within SEA workflows.

**Table 3:** Vignette challenges, successes, and lessons learned.

Lessons learned	Challenges faced	Successes
Establishing an Older-Adult Care Champion role streamlined access to training and enabled safety-and-quality flags development.	Expanding the champion role across multiple clinics required extensive coordination to maintain consistency in role functions.	Noticeable improvements in medication adherence among older adults.
Rapid-cycle motivational skills training revealed the need for ongoing adaptation to meet diverse clinical contexts.	Resistance emerged when scaling interdisciplinary training across all sites, necessitating iterative adjustments.	Reduced hospitalization rates through enhanced medication safety and coordinated teamwork.
Implementing SEA assessment training underscored the importance of comprehensive education for all staff.	Ensuring timely, consistent dissemination of annual Beers List updates posed logistical challenges.	Clinical champions successfully delivered annual Beers List updates, improving prescribing practices.
Developing annual clinic quality metrics highlighted the value of continuous performance monitoring.		Enhanced interprofessional collaboration and accountability through shared metric reporting.

SEA, Safety, Efficacy, and Adherence.

### 4.1 Practice-based monitoring and equity-responsive implementation of the SEA model

The SEA (Safety, Efficacy, and Adherence) model uses *practice-embedded monitoring* to minimize extra workload for patients and staff. During pharmacist safety rounds, adverse drug events (ADEs) are automatically logged and mapped to International Classification of Diseases, 10th Revision (ICD-10) codes. Nursing intake screens capture adherence with the 8-item Morisky Medication Adherence Scale (MMAS-8) (49), while electronic medical-record (EMR) dashboards display real-time counts of polypharmacy cases and Beers-listed medications (18).

Each clinic then follows Standards for Quality Improvement Reporting Excellence 2.0 guidance (87): defining its context, bundling interventions (e.g., safety-review cadence, deprescribing huddles (38), adherence coaching (48)), and tracking outcomes with monthly statistical-process-control run charts. Data are fed back to teams through 30-day Plan–Do–Study–Act (PDSA) cycles (83). Because all metrics are woven into existing workflows, clinics—even resource-constrained safety-net settings—can monitor success without additional staff (37).

#### 4.1.1 Public health and equity significance

Medication-related harm is inequitably distributed across populations, with intersectional factors such as age, race, and socioeconomic status contributing to elevated risk among marginalized groups. Available monitoring data suggest that low-income older adults of color experience disproportionately higher rates of activities of daily living (ADE) compared to higher-income White older adults (27, 55). These disparities continue despite efforts to improve healthcare quality (88), highlighting the persistent influence of structural inequities such as limited access to care, systemic bias, and chronic underinvestment in community health resources. Addressing these inequities calls for medication management strategies that intentionally incorporate SDOH and focus on upstream drivers of medication-related risk.

The SEA model seeks to integrate pharmacist-led safety reviews with culturally responsive adherence support as part of a broader strategy to help narrow these disparities. Equity success is defined within the model as achieving measurable reductions in medication-related risks across Area Deprivation Index quintiles over 12 months. While formal studies have yet to establish the precise cost-effectiveness of this approach, preliminary modeling suggests that SEA may offer value consistent with commonly accepted thresholds for healthcare interventions (33). By utilizing existing reimbursement mechanisms SEA is designed to promote financial sustainability within current payment structures and potentially reducing reliance on external grant funding. This positions SEA as a scalable public health intervention that aligns with the WHO Healthy Aging framework (27) for reducing medication-related harm globally.

To illustrate SEA application at the patient level, the following section presents a case description. This narrative offers a human-centered view of how the model supports safety, efficacy, and adherence in the complex care of older adults.

### 4.2 Patient-level application: a composite case example

This illustrative vignette is a composite case, synthesized from themes and intervention patterns observed across multiple SEA-aligned care settings. It does not reflect a real individual but exemplifies the patient’s journey under SEA-informed care.

#### 4.2.1 Case overview

Ms. A is a 78-year-old Filipina woman living independently in a subsidized senior apartment complex in a high-density, under-resourced neighborhood. She receives primary care at a safety-net clinic where she has been an intermittent patient for over a decade. Her medical history includes type 2 diabetes, hypertension, osteoarthritis, and mild cognitive impairment. Ms. A’s medication list includes 11 prescriptions—three of which are on the Beers list, including a long-acting sulfonylurea and benzodiazepine prescribed for insomnia (18). Over the past year, she has experienced two falls, one of which resulted in a brief hospitalization. She discontinued two medications, citing unclear instructions and fear of unwanted effects. Her daughter, who lives 90 min away, provides occasional support but is not involved in her routine care.

During a routine primary care visit, the intake nurse administered standardized screening tools that triggered SEA (Safety, Efficacy, and Adherence) protocols. The Mini-Cog flagged mild cognitive impairment (score: 2/5), while the MMAS-8 (8-item Morisky Medication Adherence Scale) yielded a score of 3/8, indicating low medication adherence. In parallel, the care team used a structured SDOH (Social Determinants of Health) screening tool—such as the Accountable Health Communities (AHC) screener —which identified financial hardship, transportation barriers, and social isolation as key risk factors.

These findings were documented in the electronic medical record (EMR), triggering a closed-loop team communication via the embedded care coordination messaging system. This closed-loop communication, a core IBH strategy, ensures that each care team member receives timely, actionable information and confirms follow-through.

In response, a pharmacist-led medication safety review flagged three high-risk medications, including a long-acting sulfonylurea (glyburide) and a benzodiazepine, both of which are identified on the Beers Criteria as potentially inappropriate for older adults due to increased risks of falls, hypoglycemia, and cognitive impairment. Recognizing these risks, the interprofessional team—comprising the primary care provider (PCP), pharmacist, behavioral health clinician, and care manager—convened a deprescribing huddle.

The pharmacist recommended substituting glyburide with a shorter-acting agent such as glipizide to reduce the likelihood of hypoglycemia. The behavioral health clinician—integrated into the primary care team—provided motivational interviewing-based coaching to assess readiness for change and explore potential adherence barriers. Meanwhile, the care manager initiated referrals through a community resource platform (e.g., Unite Us or FindHelp.org) to address identified SDOH needs.

This team-based intervention illustrates core IBH principles: coordinated care across disciplines, real-time data use to guide decision-making, and alignment of treatment with patient-centered goals. Through SEA’s structured workflows, medication safety and adherence were addressed simultaneously with cognitive and social risk factors—offering a coherent, scalable model for improving outcomes in complex care populations.

Using the PACS acronym to guide the process her case manager reviewed Ms. A’s recent encounters to identify concrete adherence barriers. By applying the four domains—People (caregiver changes), Access (transport issues), Commitment (doubts about treatment), and Systems (lack of reminders)—the team crafted a personalized plan.

This process revealed critical gaps across all domains. In the People domain, Ms. A’s limited social support was addressed by engaging her daughter and connecting her to local community-based aging services. Under Access, the team arranged pharmacy delivery services and facilitated enrollment in financial assistance programs to address cost and transportation barriers (55). The behavioral health clinician advanced the Commitment domain by using culturally responsive education and motivational interviewing (48) to explore Ms. A’s health beliefs and medication hesitancy. Within the Systems domain, the team provided adherence supports like a simplified organizer and reminders to reduce complexity and support self-management.

Together, these integrated strategies established a foundation for the clinical and functional improvements observed over the following months.

#### 4.2.2 Clinical and functional outcomes

Over the next 3 months, Ms. A’s medication regimen was reduced from 11 to 8 prescriptions. She reported improved sleep and fewer episodes of dizziness. Blood pressure and HbA1c readings moved closer to target levels. Her medication adherence score improved from 3 to 6 (49). With the care manager’s assistance, Ms. A was connected to a local meal delivery program, reducing her need to shop alone. At 6 months, she reported fewer missed doses, improved energy, and greater confidence in managing her medications.

#### 4.2.3 Equity and systems review and intervention

This composite case illustrates how SEA’s team-based framework incorporates equity considerations into care planning for older adults from historically marginalized communities. Ms. A’s profile—a 78-year-old Filipina woman living on low income in a subsidized senior complex—reflects intersecting vulnerabilities of age, gender, ethnicity, limited English proficiency, financial strain, and social isolation, each of which may increase her risk for adverse medication outcomes (27, 55).

From intake onward, the nursing team extended standard SDOH screening using the PRAPARE tool (72), supplementing it with elements of the Cultural Formulation Interview (87). These assessments identified Ms. A’s preference for Tagalog, her use of traditional herbal remedies for diabetes, and her weekly engagement with a local church community. These insights suggested that unclear translated instructions and mistrust of unfamiliar medications had contributed to her prior self-discontinuation of two prescriptions.

During the interprofessional deprescribing huddle (38), the team adopted a culturally responsive approach by partnering with a Filipino community health worker to co-facilitate a family education call in Tagalog. This approach respected Ms. A’s preference for collective decision-making and ensured accurate dosing information. Recognizing the importance of faith and community in her support network, the team connected her with a senior ministry that provided grocery delivery and peer support for chronic disease management. Educational materials were co-developed using culturally relevant metaphors—e.g., balancing blood sugar by arranging ingredients in a traditional pancit dish—and tailored to her health literacy level.

At the systems level, the team identified structural inequities that had limited Ms. A’s access to follow-up care—including clinic hours that conflicted with her daughter’s work schedule and inconsistent interpreter availability. These barriers reflected broader patterns of institutional racism, ageism, and bias related to socioeconomic status that often affect older adults from marginalized communities. In response, the team advocated for expanded interpreter coverage during afternoon clinics (88), arranged mobile pharmacy delivery through the county’s Aging and Adult Services, and flagged Ms. A’s chart to ensure continued support for navigating cultural, linguistic, and systemic barriers to care.

**Figure 3:**
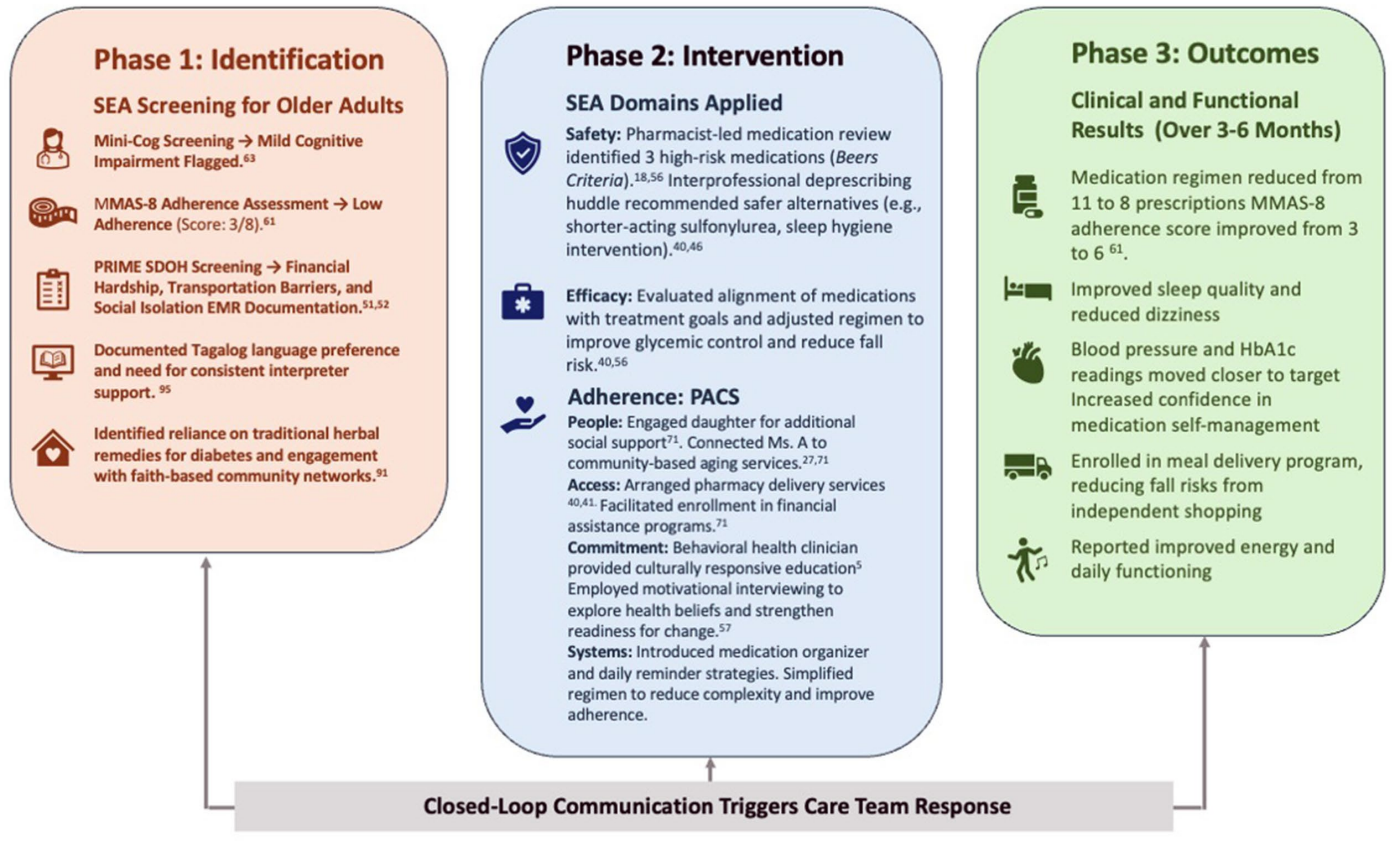
SEA patient journey. To demonstrate how the SEA model can be operationalized in a real-world health system, this section presents a constructed example of organizational implementation across a community-based network. While the case is illustrative, it synthesizes insights from multiple real-world quality improvement efforts and reflects recurring implementation strategies in FQHCs serving aging, medically complex populations (25, 30). EMR, electronic medical record; FQHC, Federally Qualified Health Center; HbA1c, glycated hemoglobin; MMAS-8, Morisky Medication Adherence Scale, 8-item version; SDOH, social determinants of health; SEA, Safety, Efficacy, and Adherence.

By integrating culturally tailored education, community partnerships, and structural advocacy, the SEA model supports individualized care that extends beyond standard protocols. Ms. A’s reports improvements in medication adherence, clinical outcomes, confidence in managing her health, and quality of life (Figure 3).

The team coordinated brief check-ins with Ms. A’s caregiver’s existing schedule, aligning visits with her daughter’s availability. This reduced missed appointments and improved continuity. To address the structural barriers that hinder equitable care delivery, SEA implementation emphasized systems-level interventions alongside individualized strategies.

Interdisciplinary collaboration was strengthened through codified workflows and clear role delineations. Pharmacists, empowered by routine access to EMR-integrated alerts, pre-visit reviews to flag polypharmacy risks. Nurses focused on reconciliation tasks, while behavioral health clinicians leveraged motivational interviewing techniques to enhance medication adherence. Care managers coordinated social risk interventions, linking patients with community resources such as meal deliveries and peer support groups.

By bridging the silos that previously fragmented care pathways, SEA’s phased rollout created a cohesive network of support. This structural alignment, coupled with dynamic training sessions and live tracking dashboards, enabled clinics to proactively address gaps in adherence support and deprescribing practices. The results were evident in improved clinical outcomes, streamlined workflows, and increased confidence among both patients and clinicians in achieving sustainable health management.

The SEA model offers a stable, equity-focused approach to caring for older adults with complex health and social needs. By aligning workflows with patient values, it embeds equity into care planning. Partnerships with cultural navigators and health ministries helped ensure interventions reflected lived experience and addressed structural drivers. Clinics used equity-informed tools to screen for social needs, identify barriers like racism and ageism, and deliver responsive care. Structured workflows, such as deprescribing huddles, bridged medical care and social action to improve outcomes and quality of life.

### 4.3 Illustrative vignette: a mock case of system-level change

The simulated network includes 14 primary care clinics serving approximately 42,000 patients annually, with 38% of the population aged 60 or older. Before implementing SEA, nearly half (47%) of older adults were prescribed five or more medications (13). Pharmacist involvement in medication safety was limited, and deprescribing was handled inconsistently (38, 44). Support for medication adherence varied across clinics, and quality improvement lacked consistent infrastructure (39, 80). These challenges were especially pronounced among older adults facing cognitive decline, housing instability, or co-occurring mental health conditions (72).

SEA was introduced using a phased Plan–Do–Study–Act (PDSA) framework over 24 months. Implementation was guided by the Consolidated Framework for Implementation Research (CFIR) and occurred in four quarterly cohorts (39, 80, 81). Each clinic designated a clinical champion—such as a pharmacist, primary care provider, or behavioral health clinician—to lead local adaptation (20, 28). Core strategies included EMR-integrated Beers Criteria alerts (18), daily deprescribing huddles (38, 44), and structured adherence screening at nurse intake (49).

A rapid diagnostic using the 4Ts framework identified several system gaps. Fewer than 25% of prescribers—and only two pharmacists—had received geriatrics or deprescribing training in the past 5 years. Nurses used the MMAS-8 without formal instruction. Based on these findings, SEA orientation included updated Beers Criteria training (18) and motivational interviewing practice (48).

Workflow mapping showed that medication review, adherence counseling, and social risk screening occurred in silos. Pharmacists reviewed charts post-visit, behavioral health clinicians followed up weeks later, and care manager referrals were inconsistent. To address this, SEA created a unified huddle template linking pharmacist chart reviews, MMAS-8 prompts, and referral checklists (49).

Teams also lacked access to real-time quality data. Metrics were only shared quarterly, limiting responsiveness (18). SEA introduced live run charts, clinic dashboards, and quarterly learning sessions to support data-driven improvement (80, 83).

Team interviews uncovered role ambiguity. Physicians expected pharmacists to deprescribe, while pharmacists lacked authority. Care managers were unsure when to engage social supports (20, 28). In response, SEA clarified roles: pharmacists handled pre-visit reviews, behavioral health clinicians provided motivational interviewing (48), care managers coordinated social follow-up, and nurses addressed flagged medications (18).

To model financial impact, SEA drew from outcomes in similar real-world programs. Pharmacist-led deprescribing, using Beers Criteria, has been associated with $684 to $1,500 in annual savings per older adult (18). Integrated Behavioral Health models have shown reductions in hospitalizations (11%), emergency visits (23%), and total medical costs by up to 10% (48, 49). Coordinated care for high-risk patients—including pharmacists, behavioral health providers, and care managers—has saved between $250 and $1,100 per patient annually (20, 28, 38). While these projections are not derived from direct SEA financial data, they reflect the plausible cost benefits of a structured, team-based approach within value-based care models.

By directly linking each diagnostic insight to a concrete design element—orientation training, the huddle workflow, real-time dashboards, and clarified roles—the 4Ts assessment provided the logic that shaped the phased interventions and underpinned the system-level gains reported over the 24-month implementation.

#### 4.3.1 Simulated example: SEA integration timeline and milestones

Implementation unfolded over 24 months through four overlapping phases. The initial 6 months focused on foundation building: teams completed SEA orientation, electronic records began firing real-time Beers alerts at prescribing (18), and clinics hardwired daily deprescribing huddles. Two early adopter clinics then served as live laboratories, using PDSA cycles to refine pharmacist polypharmacy reviews, behavioral health motivational interviewing touchpoints (48), and care manager referral pathways (49, 83). With workflows tested, remaining clinics adopted the full package, and quarterly CFIR-guided learning sessions accelerated the diffusion of successful tactics (81).

The final 6 months emphasized sustainability: huddle leadership rotated among trained champions (20, 28), audit dashboards tracked run chart trends (80), and motivational interviewing “booster” visits addressed adherence plateaus (48). SEA logic became routine, with PDSA cycles continuing autonomously (80). Pharmacists used updated Beers Criteria to review charts; behavioral health clinicians tracked adherence scores and applied motivational interviewing; care managers screened for isolation (Lubben Social Network Scale) and coordinated community or nursing support (18, 48, 49, 70).

By month 12, the percentage of older adults with Beers-listed prescriptions dropped from 28 to 17% (18), MMAS-8 scores (adherence) rose from 58.4 to 67.6 (49), polypharmacy rates fell by 12% (13), and 30-d ADE readmissions declined from 6.2 to 3.9 per thousand encounters. These gains held steady through year 2, demonstrating how a structured learning system converted phased implementation into durable safety, efficacy, and adherence improvements.

**Figure 4:**
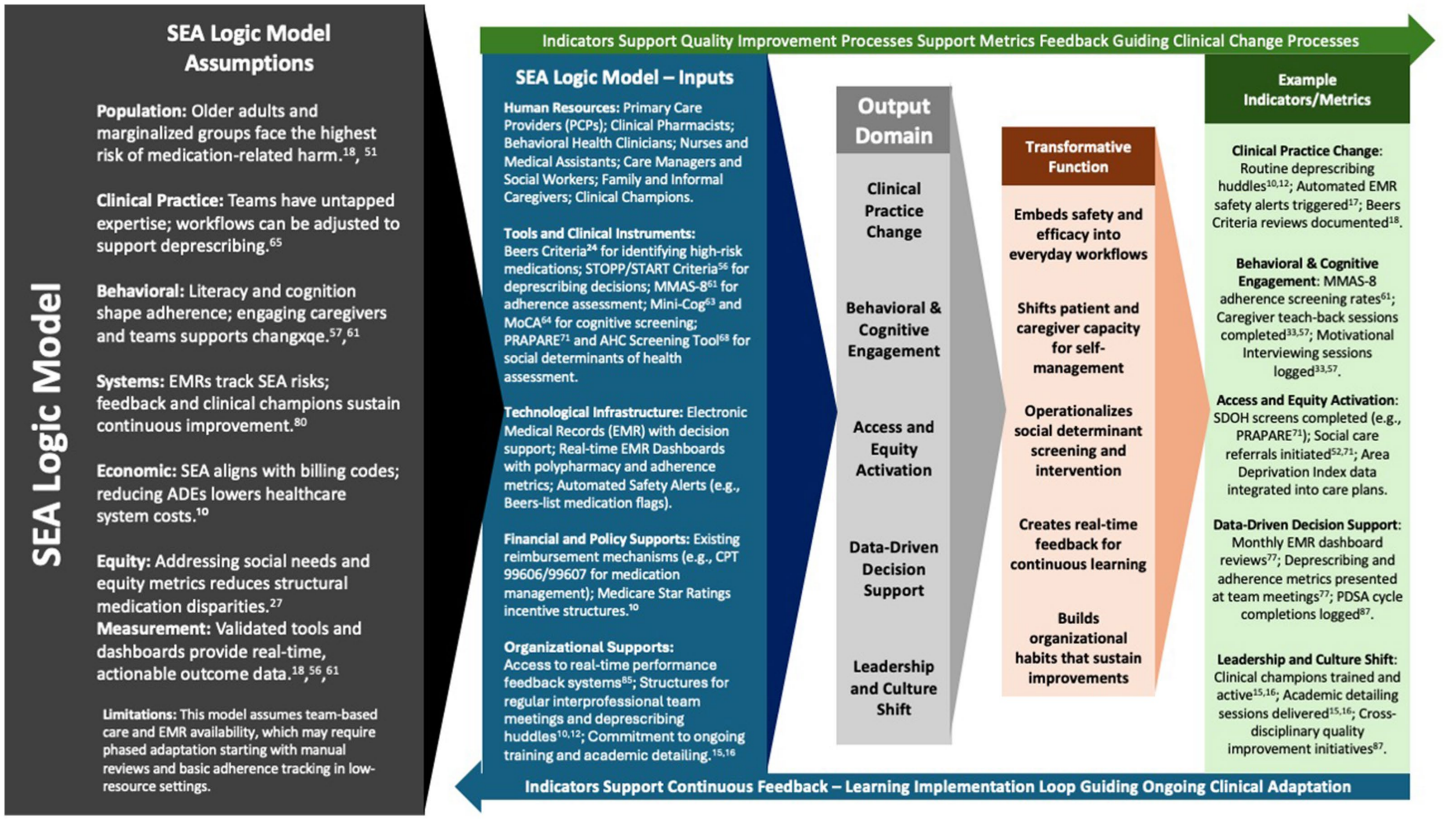
SEA logic model: assumptions, inputs, and transformative process domains supporting adaptive systems change. This logic model illustrates how the SEA framework translates foundational assumptions and implementation inputs into transformative clinical processes and measurable outputs. The model emphasizes a continuous learning feedback loop, where indicators guide real-time evaluation and adaptive clinical improvement. Equity, behavioral engagement, and leadership sustainability are positioned as core drivers of long-term systems change. ADE, adverse drug event; AHC, Accountable Health Communities; CPT, Current Procedural Terminology; EMR, electronic medical record; MMAS-8, Morisky Medication Adherence Scale, 8-item version; MoCA, Montreal Cognitive Assessment; PCP, primary care provider; PDSA, Plan–Do–Study–Act; PRAPARE, Protocol for Responding to and Assessing Patients’ Assets, Risks, and Experiences; SDOH, social determinants of health; SEA, Safety, Efficacy, and Adherence; STOPP/START, Screening Tool of Older Persons’ Prescriptions/Screening Tool to Alert to Right Treatment.

These simulated quality improvement outcomes correspond to the SEA model’s three core domains: Safety (reductions in Beers-listed medications and ADE-related readmissions) (18), Efficacy (decline in polypharmacy rates) (13), and Adherence (MMAS-8 improvement) (49). Table 4 illustrates how embedded monitoring tools and phased implementation strategies can yield measurable system-level gains in primary care environments (80, 81). Metrics were selected for alignment with the SEA logic model (Figure 4) and the public health objective of reducing medication-related harm while improving clinical outcomes and health equity for aging populations.

**Table 4:** Simulated outcomes following SEA model implementation in an FQHC network.

Outcome metric	Baseline (Q1, Year 1)	Post-implementation (Q4, Year 2)	Suggested citations
Proportion of older adults prescribed ≥1 Beers-listed medication	28%	17%	AGS Beers Criteria (18)
Median number of deprescribing huddles per site per month	0	5	D-PRESCRIBE trial (38), OPTIMIZE trial (44)
Mean MMAS-8 score among screened patients	58.4	67.6	MMAS-8 validation (49)
Polypharmacy rate (≥5 concurrent medications)	47%	35%	Polypharmacy trends (13)
30-day hospital readmission rate due to ADEs	6.2 per 1,000	3.9 per 1,000	ADE reduction from deprescribing (38), CFIR/Implementation Frameworks (81)

Data are simulated and provided for illustrative purposes only. Metrics are based on constructs from previously validated tools and implementation research. ADE, adverse drug event; AGS, American Geriatrics Society; CFIR, Consolidated Framework for Implementation Research; FQHC, Federally Qualified Health Center; MMAS-8, Morisky Medication Adherence Scale, 8-item version; SEA, Safety, Efficacy, and Adherence.

**Figure 5:**
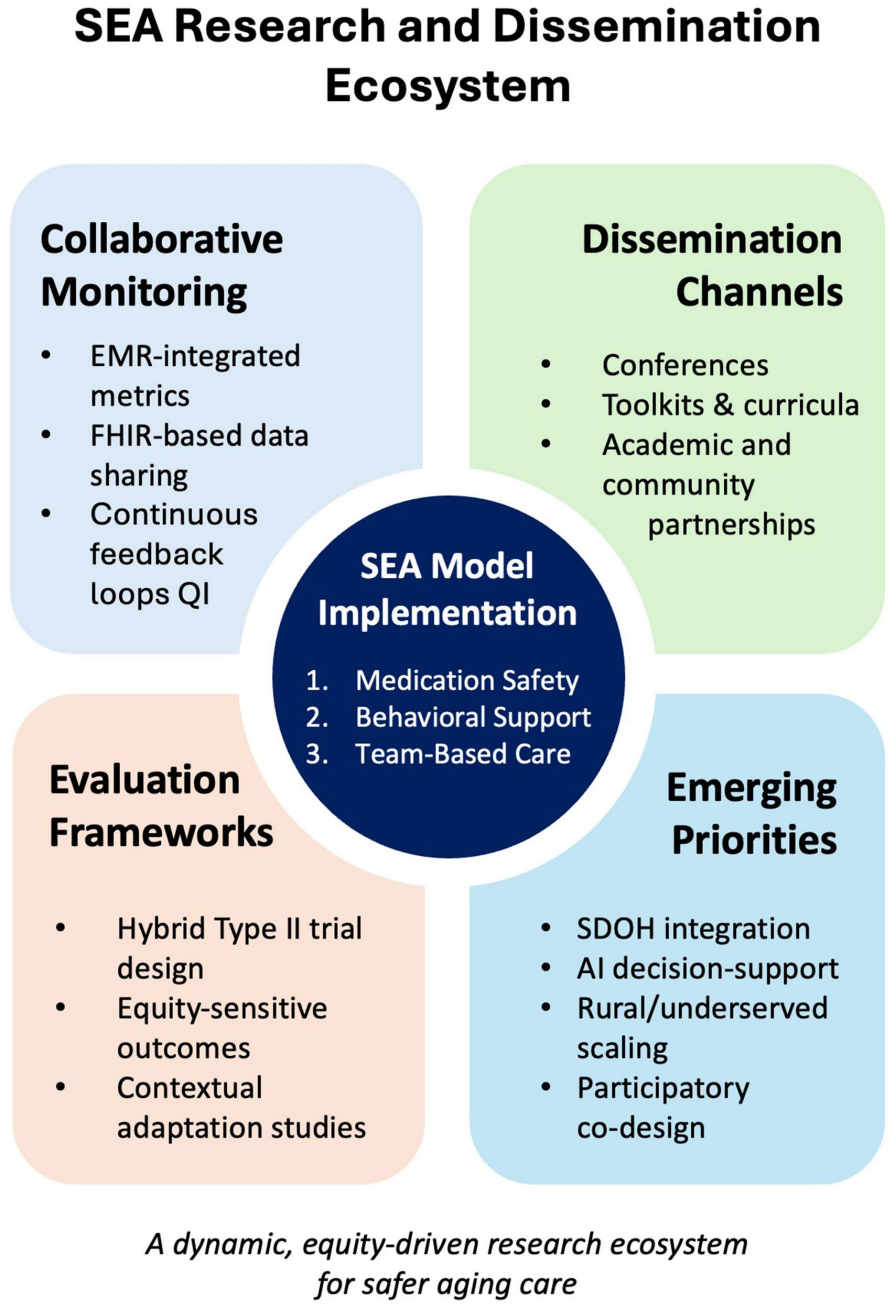
SEA research and dissemination framework: aligning evaluation, equity, and system learning. This schematic illustrates four interconnected domains that constitute a dynamic, equity-driven research ecosystem for implementing the SEA model: (1) Collaborative Monitoring—leveraging EMR-integrated metrics and FHIR-enabled data sharing to support real-time, disaggregated equity tracking (86); (2) Dissemination Channels—utilizing conferences, open-access toolkits, and academic–community partnerships to accelerate uptake across diverse settings (42, 63); (3) Evaluation Frameworks—applying hybrid Type II trial designs and equity-sensitive outcome measures to simultaneously assess clinical effectiveness and implementation fidelity (18, 39, 94); and (4) Emerging Research Priorities—focusing on the integration of SDOH, artificial intelligence decision-support, scaling in rural and underserved contexts, and participatory co-design methods to enhance responsiveness and sustainability (22, 55, 94). Together, these components create a continuous learning loop essential for sustaining and scaling safer aging care. Abbreviations: AI = artificial intelligence; EMR, electronic medical record; FHIR, Fast Healthcare Interoperability Resources; SDOH, social determinants of health; SEA, Safety, Efficacy, and Adherence.

This illustrative vignette synthesizes implementation strategies from primary care networks (25, 30) to reflect evidence-based practices and demonstrate how SEA principles can be applied in community settings (81). It highlights structured team roles (20, 28), embedded decision support (18), phased deployment (80), billing alignment (38), and integration into quality improvement—offering a scalable path aligned with the SEA logic model (Figure 5).

### 4.4 Workforce and curriculum integration: building capacity for SEA implementation

This section outlines how the SEA model aligns with public health workforce competencies and geriatric education frameworks to ensure scalable and sustainable implementation (14, 25). The 4Ts approach organizes the necessary infrastructure for embedding SEA into both clinical care and professional development systems.

#### 4.4.1 Core competency alignment: iCCOA and public health training domains

SEA (Safety, Efficacy, and Adherence) implementation depends on interprofessional collaboration and systems-based practice, mapping directly onto all eight domains of the Interprofessional Curriculum for the Care of Older Adults (iCCOA) (35). These domains include person-centered care, evidence-based practice (EBP), population health, and team functioning. SEA training therefore emphasizes medication safety (18), deprescribing principles (38, 44), adherence coaching (49), culturally responsive engagement (48), and core skills in geriatric pharmacology and health-equity–oriented care delivery (56). IBH clinicians play a central role by delivering motivational-interviewing interventions and reinforcing trauma-informed, culturally safe communication.

To align with public-health priorities, SEA curricula also incorporate the Core Competencies for Public Health Professionals, such as analytical assessment, policy development, and cultural competence (56). This dual mapping allows SEA to serve not only as a clinical training program but also as a workforce-development strategy that expands system-level capacity for an aging population. By grounding training in both interprofessional treatment and public-health competencies, SEA bridges frontline clinical practice and larger equity goals.

#### 4.4.2 Training infrastructure and champion development

Training begins with onboarding modules and experiential learning sessions tailored to each role—physicians, pharmacists, behavioral health clinicians, nurses, and care managers. Using case-based learning and role-play, learners develop SEA competencies through real-world scenarios. Interprofessional case conferences and deprescribing huddles are introduced as core components of clinical education (14, 28).

Clinical champions—experienced team members who model SEA practices and support their peers—play a key role in implementation (20, 28). A structured champion pathway includes observation, mentorship, facilitated PDSA cycles (80), and ongoing peer coaching. These champions also serve as liaisons between clinic leadership and frontline staff, ensuring feedback loops and workflow adaptation to local context (75, 84).

The SEA model operationalizes its educational goals through the 4Ts framework. Training ensures that all team members understand the SEA model, their roles, and tools used for screening, deprescribing, and adherence coaching (14). Treatment Pathways define clinical protocols that embed SEA principles into care delivery—e.g., standardizing Beers Criteria screening at intake (18) or linking MoCA results to pharmacist reviews (50). Tracking Metrics connects routine clinical work to outcome monitoring, using EMR dashboards and team feedback reports to measure adherence (49), medication risks (18), and functional outcomes (66). Team-Based Care reinforces interprofessional integration, ensuring that each SEA function is shared across a coordinated team structure (20, 28).

These domains track quality metrics aligned with CMS and HRSA—such as adherence, polypharmacy, and adverse drug event (ADE) reduction (79)—and are embedded in continuing education, onboarding, and academic detailing (15, 38). Table 5 links the 4Ts to implementation and workforce development.

**Table 5:** Role-specific SEA curriculum within the 4Ts framework.

Team member	Training	Treatment	Tracking metrics	Team-based care
Primary care provider	Deprescribing, efficacy reviews	Final med plan approval	Clinical outcomes (BP, A1c)	Leads huddles, aligns care goals
Pharmacist	ADE identification, Beers Criteria	Medication review, taper plans	Drug interaction logs, ADE flags	Educates team, flags polypharmacy
Behavioral health clinician	MI for adherence, trauma-informed care	Patient readiness, psychoeducation	MMAS-8 tracking	Prepares patient, caregiver coaching
Nurse/MA	Safety screeners, fall risk	Early symptom tracking	Intake data, EMR flags	Real-time team coordination
Care manager/SW	SDOH tools, CLAS training	Access planning, caregiver liaison	Lubben, AHC screener tracking	Closes gaps, manages follow-up

Each column corresponds to one of the 4 Ts domains and shows how targeted competencies are assigned to primary care providers, pharmacists, behavioral health clinicians, nurses/medical assistants, and care managers. The mapping links SEA training to day-to-day workflows and equity-responsive quality measures (e.g., SDOH tracking), creating a template that sites can adapt for onboarding, continuing education, and performance evaluation (18, 20, 28, 48, 49, 70). A1c, glycated hemoglobin; ADE, adverse drug event; AHC, Accountable Health Communities; BP, blood pressure; CLAS, Culturally and Linguistically Appropriate Services; EMR, electronic medical record; MA, medical assistant; MI, Motivational Interviewing; MMAS-8, Morisky Medication Adherence Scale, 8-item version; SDOH, social determinants of health; SW, social worker. Adapted from: American Geriatrics Society Beers Criteria (18), Morisky MMAS-8 adherence scale (49), Lubben Social Network Scale (70), Motivational Interviewing (48), and implementation science sources (14, 20, 28).

#### 4.4.3 Policy levers, reimbursement pathways, and sustainability planning

SEA-aligned activities are supported by several established funding and reimbursement pathways. At the federal level, the Health Resources and Services Administration (HRSA) Geriatric Workforce Enhancement Program funds interprofessional training aligned with SEA’s focus on deprescribing, adherence, and systems-based care (11, 20). On the reimbursement side, Medicare’s Medication Therapy Management (MTM) billing codes—Current Procedural Terminology (CPT) 99,605 (initial), 99,606 (established), and 99,607 (add-on time)—offer direct payment for pharmacist-led safety reviews that are central to SEA implementation (26).

Medication adherence is also incentivized under Medicare Part D Star Ratings, which reward performance on conditions such as hypertension, diabetes, and hyperlipidemia. Improvements in medication adherence scores—through SEA’s tracking and Team components—can raise ratings and secure financial bonuses (76). For health systems participating in value-based care under the Medicare Access and CHIP Reauthorization Act (MACRA) or Merit-based Incentive Payment System (MIPS), SEA activities may qualify as Improvement Activities, supporting payment adjustments and clinical quality gains (77, 79).

To support long-term sustainability, SEA is designed for integration into maintenance of certification, continuing medical education (CME), and quality dashboards. Annual updates to the Beers Criteria inform deprescribing huddles, while quarterly run chart reviews satisfy Joint Commission and governance requirements (18, 43). Modular academic detailing allows quick updates without major curriculum changes (16, 89). This policy-aligned design enables SEA to scale across systems, align with incentive structures, and address national concerns about workforce shortages and polypharmacy—while maintaining model fidelity.

#### 4.4.4 Equity-responsive and contextual adaptation

SEA’s workforce development agenda is intentionally designed to be equity-responsive (35, 90). Training modules incorporate the U. S. Office of Minority Health’s Culturally and Linguistically Appropriate Services standards and teach trauma-informed medication counseling to address structural barriers that often disproportionately affect historically underserved populations (88). Learners engage in plain language communication, health literacy “teach-back,” and the use of interpreters, applying these skills thoughtfully within SEA’s Treatment and Team domains (68, 75). Concurrently, the model integrates SDOH screening tools—such as the AHC questionnaire—to support pharmacists and care managers in connecting medication plans to patients’ transportation, housing, and food security needs (69, 72).

Clinical champions are prepared to adapt SEA workflows to resource-constrained settings—including rural clinics and Federally Qualified Health Centers (FQHCs) where staffing ratios and broadband access may differ from larger systems (20, 28). Adaptation checklists encourage teams to prioritize essential SEA components (e.g., Beers alerts, deprescribing huddles) and gradually incorporate optional elements as local capacity allows, promoting ongoing feasibility and meaningful impact in diverse care environments (80, 81).

#### 4.4.5 Educational evaluation and learner feedback

SEA training is evaluated with a multi-tiered strategy aligned with Kirkpatrick’s outcomes framework and Miller’s pyramid of clinical competence. Level 1 reaction and Level 2 knowledge gains are captured through pre−/post-tests and the SPICE-R2 interprofessional attitudes scale (14, 91, 92). Level 3 behavioral change is assessed in high-fidelity simulations that use objective structured clinical examination checklists to verify deprescribing conversations, trauma-informed counseling, and SDOH referrals (10, 93). Level 4 results appear in run chart trends—reduced Beers prescribing and improved MMAS-8 adherence scores—looped back to trainees during quarterly learning sessions. All learners maintain reflective journals, which faculty code for implementation barriers and facilitators; these qualitative insights inform rapid updates to curricular content and site-specific rollout plans (35).

These evaluation methods support continuous refinement of SEA training. While early data show gains in knowledge and behavior, ongoing assessment is needed to ensure sustained impact and equitable outcomes. Grounded in public health competencies and geriatric education standards (35, 90), this structure promotes safer, person-centered medication management. Reduced high-risk prescribing and better adherence—indicate readiness for broader implementation-science evaluation (18, 39, 42).

To sustain SEA as a learning network, we propose a multisite hybrid Type II effectiveness–implementation trial spanning three contrasting systems: (1) an FQHC consortium serving safety-net populations, (2) a Veterans Health Administration regional division, and (3) an integrated delivery system such as Kaiser Permanente (94). This design—or a stepped wedge cluster variant—would capture clinical impact and implementation fidelity simultaneously, yielding high-leverage data for scale-up decisions.

Primary outcomes would include changes in ADE incidence, adherence scores, and deprescribing frequency. Stratified analyses will assess equity-related outcomes, including shifts in ADE risk by Area Deprivation Index quintiles. While this trial has not yet been launched, the study design leverages routine SEA metrics—providing a scalable foundation for future evaluation once partnerships and funding are secured (18, 22, 33).

#### 4.4.6 Data infrastructure and collaborative monitoring

SEA implementation depends on low-burden, high-utility data systems. Many SEA-compatible clinics already collect relevant indicators—e.g., ADE counts, Beers Criteria flags, and adherence scores—within existing electronic health records (81). These systems can be augmented using FHIR-compliant APIs, EMR dashboards, and shared de-identified repositories.

A platform such as the Dryad Digital Repository could support transparent benchmarking while protecting privacy (95). Incorporating disaggregated equity metrics (e.g., by race, language, and neighborhood disadvantage) allows SEA teams to monitor disparities longitudinally. Implementation monitoring can draw from established frameworks such as RE-AIM and equity-focused approaches to guide evaluation design (28, 81).

### 4.5 Dissemination strategies

To facilitate broad adoption, dissemination efforts must extend beyond academic publications. Key strategies include conference presentations (e.g., American Public Health Association, Gerontological Society of America), partnerships with primary care collaboratives, and regional learning networks. Open-access toolkits, training curricula, and digital implementation guides will support uptake across resource-diverse settings (42).

Academic detailing, quality improvement collaboratives, and clinical champion mentoring can enable local adaptation. Partnerships with public health departments, Area Agencies on Aging, and academic-community alliances will be critical to reach underserved and aging-focused sectors.

#### 4.5.1 Research priorities emerging from implementation

As SEA is implemented across diverse contexts, several pressing research questions have emerged. First, the influence of social determinant interventions—including housing support, transportation access, and food security—on medication adherence and clinical outcomes warrants further study (20, 55). Evaluating how these supports interface with SEA’s Adherence domain enhance the integration of health-related social needs interventions.

Second, artificial intelligence tools may complement SEA by identifying deprescribing candidates or polypharmacy risks. Studies are needed to evaluate integration with clinical judgment and patient-centered care. Third, scaling SEA in under-resourced settings requires thoughtful adaptation. Mixed-methods designs can support context-responsive fidelity (18, 33).

Finally, participatory research approaches hold strong potential for advancing equity in geriatric care. By engaging older adults and caregivers—particularly those impacted by systemic racism, anti-Latiné bias, ageism, sexism, and economic hardship—in the co-design of SEA processes, teams can uncover unmet needs, cultural priorities, and communication preferences that traditional strategies may miss. These community-engaged methods bring lived experience to the center of care design, helping to identify and address barriers rooted in structural oppression. In doing so, they support the creation of more culturally responsive, trust-building services and strengthen the relevance and equity of SEA model adaptations in real-world settings.

These research directions reflect the evolution of the SEA model from a clinical quality intervention to a dynamic, equity-centered implementation framework. Continued inquiry will deepen the empirical base, support model refinement, and reinforce SEA’s alignment with public health values of safety, access, and community participation.

Altogether, the proposed trial, data infrastructure roadmap, and dissemination strategies position SEA for the level of rigorous testing and iterative refinement expected of a maturing public health innovation (39, 81, 94). Demonstrating effectiveness across diverse delivery systems, embedding equity-sensitive metrics, and cultivating a community of practice will generate the evidence and momentum required for national uptake (35, 75). The concluding section now distills the overarching implications of this work and how stakeholders can collaborate to realize safer, more person-centered medication management for older adults at scale.

## 5 Limitations

While SEA offers a structured blueprint, it is not plug-and-play. The model assumes access to pharmacists or trained technicians, EHRs with real-time alerts, and leadership support for protected team time (28, 39, 80). In safety-net or rural settings, workforce shortages and limited informatics may delay uptake or require adaptation (20, 81). Future work should clarify core versus flexible components (e.g., Beers review vs. virtual huddles). Institutional commitment is essential, and resistance to interdisciplinary practice can slow progress (28, 81). Outcomes vary with resource levels and patient demographics (6, 33).

Equity is central: polypharmacy often coexists with food insecurity, unstable housing, or low health literacy (56, 69). Without targeted SDOH interventions, SEA’s impact may plateau. Culturally responsive strategies and community health workers are critical to reach underserved populations and sustain equitable impact (35, 72, 90). Further research should evaluate real-world outcomes, satisfaction, and scalability (17, 94).

## 6 Conclusion

The SEA model offers a comprehensive and patient-centered framework to address the complex healthcare needs of older adults. By focusing on its three core principles—safety, efficacy, and adherence—the model emphasizes the importance of interdisciplinary collaboration, targeted training, and tailored interventions to enhance the overall well-being of older adults (12, 35, 90). Integrating the SEA model into clinical care demonstrates its potential to improve medication safety, reduce hospitalization, and address challenges in the aging population (21, 22, 33). Future studies prioritize overcoming barriers to implementation by advancing healthcare provider training, tailoring interventions for diverse populations, and evaluating the long-term impact of the SEA model on health outcomes and costs (10, 39, 94).

Effective medication management for older adults can improve their healthspan, lifespan, and QOL (3, 4, 22). The SEA model holds the potential to significantly enhance effective medication treatments for older adults (35, 90). To further realize this potential, research is needed to investigate the scalability and sustainability of SEA interventions across various healthcare contexts (17, 81). Exploring nuances in interdisciplinary collaboration, the role of technology in facilitating medication safety, and the incorporation of patient preferences into care plans may contribute to the ongoing evolution of the SEA model (9, 43, 63). By addressing these research gaps, the SEA model can be refined and enhanced, ultimately advancing the quality of care for older adults and promoting healthier aging in diverse communities (35, 90).

Yet these promising signals mark only the first stage of the model’s evolution. Multi-site hybrid Type II trials, coupled with stepped wedge designs in smaller systems, are needed to confirm its impact on hard outcomes—ADEs, emergency visits, functional decline—and to establish cost-effectiveness across diverse payer environments (18, 94). Equally vital is understanding how SEA performs among populations that carry the heaviest medication burden: older adults with multimorbidity, caregivers with limited English proficiency, and individuals living in high deprivation neighborhoods (22, 55, 90). Stratified analyses and equity-centered metrics will reveal whether the model closes, rather than widens, existing health gaps (75, 90).

Finally, the next wave of research should examine how emerging technologies and patient engagement tools—AI-driven candidate identification, conversational agents for medication counseling, and SDOH dashboards—can be layered onto SEA without eroding its person-centered ethos (48, 69, 86). Collaborative learning networks, knowledge repositories, and academic community partnerships will be essential to ensure that the model remains adaptable, evidence-based, and responsive to the evolving landscape of geriatric care (42, 63, 81).
