# Supplementary material for: Enhancing primary care for older adults: the safety, efficacy, and adherence (SEA) team-based care model to reduce adverse medication outcomes
Source: Front Public Health. 2025 Aug 6;13:1453485. doi: 10.3389/fpubh.2025.1453485 (PMC12364638; doi:10.3389/fpubh.2025.1453485)
Supplement: Supplementary file 1 [file Data_Sheet_1.PDF]

Supplementary Table 1. Complete literature set informing development of the Safety, Efficacy, and Adherence (SEA) model (n = 88).

The table lists every source screened during the structured, non-systematic review that guided SEA-model construction. It is numbered 1 – 88 **independently of the main in-text reference list** (those numbers are not cited in the narrative). For each entry we provide the compressed Vancouver citation and a brief "domain tag" identifying the thematic role the source played in model design (e.g., "Deprescribing," "Interprofessional education," "SDOH screening").

A cross-walk beneath the table groups the 88 sources into 10 evidence domains—medication safety, adherence, team-based care, implementation science, integrated BH/CCM frameworks, SDOH / equity tools, quality-improvement infrastructure, CDS/EMR technology, cognitive/frailty screening, and motivational-interviewing/shared-decision strategies—showing at a glance how the review set maps onto SEA's conceptual pillars.

Abbreviations: IPE, interprofessional education; MTM, medication-therapy management; PCMH, patient-centered medical home; CCM, Chronic Care Model; BH, behavioral health; SDOH, social determinants of health; CDS, clinical-decision support; EMR, electronic medical record; MI, motivational interviewing; QI, quality improvement.

7-Domain Criterion	Reference numbers
1 Medication safety & polypharmacy	1, 6, 8, 9, 14, 16, 17, 30, 36, 40, 42, 43, 46, 49, 52, 59, 60, 69, 70, 71, 72, 73, 87
2 Medication adherence & regimen complexity	5, 7, 15, 17, 25, 29, 44, 47, 53, 57, 58, 61, 62, 84
3 Integrated chronic & behavioral health care (includes SDOH / equity tools)	18, 22, 23, 24, 27, 31, 32, 39, 47, 48, 63, 66, 74, 78, 86
4 Deprescribing frameworks	6, 8, 17, 30, 36, 42, 52, 69, 70, 72
5 Interprofessional training & team-based care	1 – 4, 10, 26, 34, 35, 37, 51, 55, 56
6 Implementation science & clinical champions	11 – 13, 19 – 21, 32, 38, 54, 79, 80, 81, 88
7 Quality measurement & improvement (includes CDS / EMR / population-health tech)	40, 41, 54, 67, 68, 75, 76, 77, 79, 80, 81, 82, 83, 87, 88

Supplementary Table 2. Criterion-to-evidence cross-walk for the SEA-model literature set.

This table maps the 88 references selected during the structured, domain-guided literature review process (illustrated in Figure 1) to the specific domains they informed during the development of the SEA model. Each row corresponds to a reference listed in Supplementary Table 1 and indicates its contribution to one or more of the following seven domains: medication safety and polypharmacy; medication adherence and regimen complexity; integrated chronic and behavioral health care; deprescribing frameworks; interprofessional training and team-based care; implementation science and clinical champions; and quality measurement and improvement. The table is designed to document the relevance and distribution of evidence sources across model domains. It does not affect in-text citation numbering in the manuscript.

Domain-code key (7-factor schema):
1 = Medication safety & polypharmacy 2 = Medication adherence 3 = Integrated chronic / behavioral-health care 4 = Deprescribing frameworks 5 = Interprofessional training & team-based care 6 = Implementation science & clinical champions 7 = Quality measurement & improvement

#	Vancouver-style reference	Domain codes
**1**	Grimes TC, Guinan EM. Interprofessional education focused on medication safety: a systematic review. J Interprof Care (2023) 37:131–49. doi: 10.1080/13561820.2021.2015301.	1, 5
**2**	Lally KM, Ducharme CM, Roach RL, Towey C, Filinson R, Tuya Fulton A. Interprofessional training: Geriatrics and palliative care principles for primary care teams in an ACO. Gerontol Geriatr Educ (2019) 40:121–31. doi: 10.1080/02701960.2018.1459595.	5
**3**	Sanders KA, Busby-Whitehead J, Coppola S, Dews D, Downey CL, Giuliani C, et al. An interprofessional experience preparing a collaborative workforce to care for older adults. Gerontol Geriatr Educ (2023) 44:339–53. doi: 10.1080/02701960.2022.2052870.	5
**4**	Schussel KE, Forbes S, Taylor AM, Cooley JH. Implementation of an interprofessional medication therapy management experience. Am J Pharm Educ (2019) 83:6584. doi: 10.5688/ajpe6584.	5, 6
**5**	Anderson LJ, Nuckols TK, Coles C, Le MM, Schnipper JL, Shane R, et al. A systematic overview of systematic reviews evaluating medication adherence interventions. Am J Health Syst Pharm (2020) 77:138–47. doi: 10.1093/ajhp/zxz284.	2
**6**	Doherty AJ, Boland P, Reed J, Clegg AJ, Stephani AM, Williams NH, et al. Barriers and facilitators to deprescribing in primary care: a systematic review. BJGP Open (2020) 4:bjgpopen20X101096. doi: 10.3399/bjgpopen20X101096.	4
**7**	Iuga AO, McGuire MJ. Adherence and health care costs. Risk Manag Healthc Policy (2014) 7:35–44. doi: 10.2147/RMHP.S19801.	2
**8**	Radcliffe E, Servin R, Cox N, Lim S, Tan QY, Howard C, et al. What makes a multidisciplinary medication review and deprescribing intervention for older	4

	people work well in primary care? A realist review and synthesis. BMC Geriatr (2023) 23:591. doi: 10.1186/s12877-023-04256-8.	
**9**	Wang X, Liu K, Shirai K, Tang C, Hu Y, Wang Y, et al. Prevalence and trends of polypharmacy in U.S. adults, 1999–2018. Glob Health Res Policy (2023) 8:25. doi: 10.1186/s41256-023-00311-4.	1
**10**	Lamparyk K, Williams AM, Robiner WN, Bruschwein HM, Ward WL. Interprofessional education: current state in psychology training. J Clin Psychol Med Settings (2022) 29:20–30. doi: 10.1007/s10880-021-09765-5.	5
**11**	Yeh JS, Van Hoof TJ, Fischer MA. Key features of academic detailing: development of an expert consensus using the Delphi method. Am Health Drug Benefits (2016) 9:42–50.	6
**12**	Kennedy AG, Regier L, Fischer MA. Educating community clinicians using principles of academic detailing in an evolving landscape. Am J Health Syst Pharm (2021) 78:80–6. doi: 10.1093/ajhp/zxaa351.	6
**13**	Moss JM, Bryan WE, Wilkerson LM, King HA, Jackson GL, Owenby RK, et al. An interdisciplinary academic detailing approach to decrease inappropriate medication prescribing by physician residents for older veterans treated in the emergency department. J Pharm Pract (2019) 32:167–74. doi: 10.1177/0897190017747424.	6
**14**	American Geriatrics Society. American Geriatrics Society updated Beers Criteria® for potentially inappropriate medication use in older adults. 2023. Available from: https://geriatricscareonline.org/ProductAbstract/american-geriatrics-society-updated-beers-criteria/CL001/?param2=search [Accessed 2024 Oct 18].	1
**15**	Krousel-Wood, M., Craig, L. S., Peacock, E., Zlotnick, E., O'Connell, S., Bradford, D., ... & Petty, R. (2021). Medication adherence: expanding the conceptual framework. *American journal of hypertension*, *34*(9), 895-909.	2, 6
**16**	Awad A, Hanna O. Potentially inappropriate medication use among geriatric patients in primary care setting: A cross-sectional study using the Beers, STOPP, FORTA and MAI criteria. PLoS One (2019) 14:e0218174. doi: 10.1371/journal.pone.0218174.	1
**17**	Ulley J, Harrop D, Ali A, Alton S, Fowler Davis S. Deprescribing interventions and their impact on medication adherence in community-dwelling older adults with polypharmacy: a systematic review. BMC Geriatr (2019) 19:15. doi: 10.1186/s12877-019-1031-4.	2, 4
**18**	Gupta M, Ali HK, Savo D, Conroy M, Wilkins KM. Integrated primary and mental health care for older adults: successes, challenges, and recommendations. Curr Geri Rep (2019) 8:137–47. doi: 10.1007/s13670-019-00285-7.	3
**19**	Miech EJ, Rattray NA, Flanagan ME, Damschroder L, Schmid AA, Damush TM. Inside help: An integrative review of champions in healthcare-related implementation. SAGE Open Med (2018) 6:2050312118773261. doi: 10.1177/2050312118773261.	6
**20**	Wood K, Giannopoulos V, Louie E, Baillie A, Uribe G, Lee KS, et al. The role of clinical champions in facilitating the use of evidence-based practice in drug and alcohol and mental health settings: A systematic review. Implement Res Pract (2020) 1:2633489520959072. doi: 10.1177/2633489520959072.	6

**21**	Morena AL, Gaias LM, Larkin C. Understanding the role of clinical champions and their impact on clinician behavior change: the need for causal pathway mechanisms. Front Health Serv (2022) 2:896885. doi: 10.3389/frhs.2022.896885.	6
**22**	American Medical Association. Behavioral Health Integration Compendium: Older Adults Practice Guide. Available from: https://www.ama-assn.org/system/files/bhi-older-adults-practice-guide.pdf [Accessed 2024 Oct 18].	3
**23**	World Health Organization. Integrated care for older people (ICOPE): Guidelines on community-level interventions to manage declines in intrinsic capacity. https://www.who.int/publications/i/item/9789241550109 [Accessed November 26, 2024].	3
**24**	Sum G, Sim SYH, Chay J, Ho SH, Ginting ML, Lim ZZB, et al. An integrated patient-centred medical home (PCMH) care model reduces prospective healthcare utilisation for community-dwelling older adults with complex needs: a matched observational study in Singapore. Int J Environ Res Public Health (2023) 20:6848. doi: 10.3390/ijerph20196848.	3
**25**	Walsh CA, Cahir C, Tecklenborg S, Byrne C, Culbertson MA, Bennett KE. The association between medication non-adherence and adverse health outcomes in ageing populations: A systematic review and meta-analysis. Br J Clin Pharmacol (2019) 85:2464–78. doi: 10.1111/bcp.14075.	2
**26**	Gallimore CE, Kline E. A survey of pharmacists in integrated care: Benefits, barriers, and facilitators of integration. Fam Syst Health (2023) 41:222–8. doi: 10.1037/fsh0000753.	5
**27**	Sporinova B, Manns B, Tonelli M, Hemmelgarn B, MacMaster F, Mitchell N, et al. Association of mental health disorders with health care utilization and costs among adults with chronic disease. JAMA Netw Open (2019) 2:e199910. doi: 10.1001/jamanetworkopen.2019.9910.	3
**28**	Fulmer T, Reuben DB, Auerbach J, Fick DM, Galambos C, Johnson KS. Actualizing better health and health care for older adults. Health Aff (Millwood) (2021) 40:219–25. doi: 10.1377/hlthaff.2020.01470.	7
**29**	Mohan A, Majd Z, Johnson ML, Essien EJ, Barner J, Serna O, et al. A Motivational interviewing intervention to improve adherence to ACEIs/ARBs among nonadherent older adults with comorbid hypertension and diabetes. Drugs Aging (2023) 40:377–90. doi: 10.1007/s40266-023-01008-6.	2
**30**	Mekonnen AB, Redley B, de Courten B, Manias E. Potentially inappropriate prescribing and its associations with health-related and system-related outcomes in hospitalized older adults: A systematic review and meta-analysis. Br J Clin Pharmacol (2021) 87:4150–72. doi: 10.1111/bcp.14870.	1
**31**	Hills WE. Behavioral health and new models of service delivery for an aging world: public/private partnerships to develop best practices of care for older adults. Med Sci Pulse (2019) 13:29–33. doi: 10.5604/01.3001.0013.1372.	3
**32**	Grudniewicz A, Gray CS, Boeckxstaens P, De Maeseneer J, Mold J. Operationalizing the Chronic Care Model with goal-oriented care. Patient (2023) 16:569–78. doi: 10.1007/s40271-023-00645-8.	3, 6

**33**	Austin M. Interprofessional curriculum for the care of older adults (iCCOA) (2019). https://nexusipe.org/informing/resource-center/interprofessional education. [Accessed October 18, 2024].	5
**34**	Spaulding EM, Marvel FA, Jacob E, Rahman A, Hansen BR, Hanyok LA, Martin SS, Han HR. Interprofessional education and collaboration among healthcare students and professionals: a systematic review and call for action. Journal of interprofessional care. 2021 Jul 4;35(4):612-21.	5
**35**	Schapmire TJ, Head BA, *et al.* Overcoming barriers to IPE in gerontology. *Adv* Schapmire TJ, Head BA, Nash WA, Yankeelov PA, Furman CD, Wright RB, et al. Overcoming barriers to interprofessional education in gerontology: the Interprofessional Curriculum for the Care of Older Adults. Adv Med Educ Pract (2018) 9:109–18. doi: 10.2147/AMEP.S149863.	5
**36**	Martin P, Tamblyn R, Benedetti A, Ahmed S, Tannenbaum C. Effect of a pharmacist-led educational intervention on inappropriate medication prescriptions in older adults: The D-PRESCRIBE randomized clinical trial. JAMA (2018) 320:1889–98. doi: 10.1001/jama.2018.16131.	4
**37**	Wagner TD, Jones MC, Salgado TM, Dixon DL. Pharmacist's role in hypertension management: a review of key randomized controlled trials. J Hum Hypertens (2020) 34:487–94. doi: 10.1038/s41371-020-0331-7.	5
**38**	Walaszek A, Schroeder M, Albrecht T, LeCaire T, Carlsson CM. Using academic detailing to enhance the knowledge, skills and attitudes of clinicians caring for patients with behavioral and psychological symptoms of dementia. Alzheimers Dem (2021) 17:e051961. doi: 10.1002/alz.051961.	6
**39**	Walker TJ, Renn BN. "Models of integrated behavioral and mental health in primary care." In: Malone ML, Boltz M, Macias Tejada J, White H, editors. Geriatrics Models of Care: Bringing 'Best Practice' to an Aging America. Cham: Springer International Publishing (2024). p. 167–75. doi: 10.1007/978-3-031-56204-4_16.	3
**40**	Kohn LT, Corrigan JM, Donaldson MS, editors. To Err Is Human: Building a Safer Health System. Washington, DC: The National Academies Press (2000). doi: 10.17226/9728.	1, 7
**41**	The Joint Commission. Reducing handoff communication failures and inequities in healthcare (2024). https://www.jcrinc.com/resources/news/2024/08/reducing-handoff-communication-failures-and-inequities-in-healthcare/ [Accessed May 14, 2025].	7
**42**	Bayliss EA, Shetterly SM, Drace ML, Norton JD, Maiyani M, Gleason KS, et al. Deprescribing education vs usual care for patients with cognitive impairment and primary care clinicians: the OPTIMIZE pragmatic cluster randomized trial. JAMA Intern Med (2022) 182:534–42. doi: 10.1001/jamainternmed.2022.0502.	4
**43**	Mangoni AA, Jackson SH. Age-related changes in pharmacokinetics and pharmacodynamics: basic principles and practical applications. Br J Clin Pharmacol (2004) 57:6–14. doi: 10.1046/j.1365-2125.2003.02007.x.	1
**44**	Krousel-Wood M, Joyce C, Holt E, Muntner P, Webber LS, Morisky DE, et al. Predictors of decline in medication adherence: results from the cohort study of medication adherence among older adults. Hypertension (2011) 58:804–10. doi: 10.1161/HYPERTENSIONAHA.111.176859	2

**45**	Sims-Gould J, Elliott J, Tong CE, Giguère A, Mallinson S, Stolee P. A national intervention to support frail older adults in primary care: a protocol for an adapted implementation framework. BMC Geriatr (2021) 21:453. doi: 10.1186/s12877-021-02395-4.	1
**46**	Praxedes MFDS, Pereira GCDS, Lima CFDM, Santos DBD, Berhends JS. Prescribing potentially inappropriate medications for the elderly according to Beers Criteria: systematic review. Cien Saude Colet (2021) 26:3209–19. doi: 10.1590/1413-81232021268.05672020.	1
**47**	Ruksakulpiwat S, Benjasirisan C, Ding K, Phianhasin L, Thorngthip S, Ajibade AD, et al. Utilizing social determinants of health model to understand barriers to medication adherence in patients with ischemic stroke: a systematic review. Patient Prefer Adherence (2023) 17:2161–74. doi: 10.2147/PPA.S420059.	2
**48**	US Department of Health and Human Services. Social determinants of health and older adults (2023). https://health.gov/our-work/national-health-initiatives/healthy-aging/social-determinants-health-and-older-adults [Accessed October 18, 2024].	7
**49**	Gray SL, Marcum ZA, Schmader KE, Hanlon JT. Update on medication use quality and safety in older adults, 2017. J Am Geriatr Soc (2018) 66:2254–8. doi: 10.1111/jgs.15665.	1
**50**	Kim ES, Tkatch R, Martin D, MacLeod S, Sandy L, Yeh C. Resilient aging: psychological well-being and social well-being as targets for the promotion of healthy aging. Gerontol Geriatr Med (2021) 7:23337214211002951. doi: 10.1177/23337214211002951.	3
**51**	Proia KK, Thota AB, Njie GJ, Finnie RK, Hopkins DP, Mukhtar Q, et al. Team-based care and improved blood pressure control: a community guide systematic review. Am J Prev Med (2014) 47:86–99. doi: 10.1016/j.amepre.2014.03.004.	5
**52**	O'Mahony D, O'Sullivan D, Byrne S, O’Connor MN, Ryan C, Gallagher P. STOPP/START criteria for potentially inappropriate prescribing in older people: version 2. Age Ageing (2015) 44:213–8. doi: 10.1093/ageing/afu145.	1
**53**	Miller WR, Rollnick S. Motivational Interviewing: Helping People Change. 3rd ed. New York: Guilford Press (2013).	2
**54**	Ozkaynak M, Unertl K, Johnson S, Brixey J, Haque SN. “Clinical workflow analysis, process redesign, and quality improvement.” In: Finnell JT, Dixon BE, editors. Clinical Informatics Study Guide: Text and Review. Cham: Springer International Publishing (2022). p. 103–18. doi: 10.1007/978-3-030-93765-2_8.	7
**55**	Pousinho S, Morgado M, Plácido AI, Roque F, Falcão A, Alves G. Clinical pharmacists´ interventions in the management of type 2 diabetes mellitus: a systematic review. Pharm Pract (Granada) (2020) 18:2000. doi: 10.18549/PharmPract.2020.3.2000.	5
**56**	Rawlinson C, Carron T, Cohidon C, Arditi C, Hong QN, Pluye P, et al. An overview of reviews on interprofessional collaboration in primary care: barriers and facilitators. Int J Integr Care (2021) 21:32. doi: 10.5334/ijic.5589.	5
**57**	Morisky DE, Ang A, Krousel-Wood M, Ward HJ. Predictive validity of a medication adherence measure in an outpatient setting. J Clin Hypertens (Greenwich) (2008) 10:348–54. doi: 10.1111/j.1751-7176.2008.07572.x.	2

**58**	Rafhi E, Al-Juhaishi M, Stupans I, Stevens JE, Park JS, Wang KN. The influence of patients' beliefs about medicines and the relationship with suboptimal medicine use in community-dwelling older adults: a systematic review of quantitative studies. Int J Clin Pharm (2024) 46:811–30. doi: 10.1007/s11096-024-01727-9.	2
**59**	Borson S, Scanlan JM, Chen P, Ganguli M. The Mini-Cog as a screen for dementia: validation in a population-based sample. J Am Geriatr Soc (2003) 51:1451–4. doi: 10.1046/j.1532-5415.2003.51465.x.	1
**60**	Nasreddine ZS, Phillips NA, Bédirian V, Charbonneau S, Whitehead V, Collin I, et al. The Montreal Cognitive Assessment, MoCA: a brief screening tool for mild cognitive impairment. J Am Geriatr Soc (2005) 53:695–9. doi: 10.1111/j.1532-5415.2005.53221.x.	1
**61**	**Agency for Healthcare Research and Quality.** The SHARE Approach: AHRQ's Shared decision making Training Toolkit. Available from: https://www.ahrq.gov/sdm/share-approach/index.html [Accessed 2024 Oct 18].	7
**62**	Rosenstock IM. Historical origins of the Health Belief Model. Health Educ Monogr (1974) 2:328–35. doi: 10.1177/109019817400200403.	2
**63**	Billioux A, Verlander K, Anthony S, Alley D. Standardized screening for health-related social needs in clinical settings: The Accountable Health Communities screening tool. 2017. Available from: https://nam.edu/wp-content/uploads/2017/05/Standardized-Screening-for-Health-Related-Social-Needs-in-Clinical-Settings.pdf [Accessed 2024 Oct 18].	7
**64**	Lubben J, Blozik E, Gillmann G, Iliffe S, von Renteln Kruse W, Beck JC, et al. Performance of an abbreviated version of the Lubben Social Network Scale among three European community-dwelling older adult populations. Gerontologist (2006) 46:503–13. doi: 10.1093/geront/46.4.503.	2
**65**	Kokorelias KM, Hayes A, Lim Fat G, Abdelhalim R, Singh H, Saragosa M, et al. Unveiling the impact and diverse contributions of champions in community initiatives for elderly individuals with chronic conditions: an in-depth examination through a scoping review. Health Soc Care Community (2024) 2024:5025234. doi: 10.1155/2024/5025234.	6
**66**	National Association of Community Health Centers (NACHC). PRAPARE: Protocol for Responding to and Assessing Patients' Assets, Risks, and Experiences. Bethesda, MD: NACHC (2019). https://www.nachc.org/research-and-data/prapare/.	7
**67**	Epic Systems Corporation. Epic Healthy Planet: Population Health Management Platform. Verona, WI: Epic Systems (2024). https://www.epic.com/software/population-health/.	7
**68**	Oracle Health (Cerner). HealtheIntent: Population Health Management Solutions. Austin, TX: Oracle Health (2024). https://www.oracle.com/industries/healthcare/.	7
**69**	Naranjo CA, Busto U, Sellers EM, Sandor P, Ruiz I, Roberts EA, et al. A method for estimating the probability of adverse drug reactions. Clin Pharmacol Ther (1981) 30:239–45. doi: 10.1038/clpt.1981.154.	1

**70**	Hanlon JT, Schmader KE, Samsa GP, Weinberger M, Uttech KM, Lewis IK, et al. A method for assessing drug therapy appropriateness. J Clin Epidemiol (1992) 45:1045–51. doi: 10.1016/0895-4356(92)90144-c.	1
**71**	Rockwood K, Song X, MacKnight C, Bergman H, Hogan DB, McDowell I, et al. A global clinical measure of fitness and frailty in elderly people. CMAJ (2005) 173:489–95. doi: 10.1503/cmaj.050051.	1
**72**	Priyadarshini R, Eerike M, Varatharajan S, Ramaswamy G, Raj GM, Cherian JJ, Rajendran P, Gunasekaran V, Rao SV, Konda VG. Assessing the Efficacy of the ARMOR Tool–Based Deprescribing Intervention for Fall Risk Reduction in Older Patients Taking Fall Risk–Increasing Drugs (DeFRID Trial): Protocol for a Randomized Controlled Trial. JMIR research protocols. 2024 Jun 11;13(1):e55638.	4
**73**	Rudolph JL, Salow MJ, Angelini MC, McGlinchey RE. The anticholinergic risk scale and anticholinergic adverse effects in older persons. Arch Intern Med (2008) 168:508–13. doi: 10.1001/archinternmed.2007.106.	1
**74**	Stormacq C, Van den Broucke S, Wosinski J. Does health literacy mediate the relationship between socioeconomic status and health disparities? Integrative review. Health promotion international. 2019 Oct 1;34(5):e1-7.	2
**75**	Centers for Medicare & Medicaid Services (CMS). Medicare Star Ratings Technical Notes. Baltimore, MD: CMS (2024). https://www.cms.gov/newsroom/fact-sheets/2024-medicare-advantage-and-part-d-star-ratings	7
**76**	National Committee for Quality Assurance (NCQA). HEDIS 2024 Volume 1: Narrative. Washington, DC: NCQA (2024).	7
**77**	National Committee for Quality Assurance (NCQA). Person-Centered Outcome Measures for Older Adults. Washington, DC: NCQA (2024).	7
**78**	Centers for Medicare & Medicaid Services (CMS). ACO REACH Model: Equity Benchmarks and Quality Metrics. Baltimore, MD: CMS (2024). https://www.cms.gov/priorities/innovation/innovation-models/aco-reach	7
**79**	Leis JA, Shojania KG. A primer on PDSA: executing plan-do-study-act cycles in practice, not just in name. BMJ Qual Saf (2017) 26:572–7. doi: 10.1136/bmjqs-2016-006245.	7
**80**	Damschroder LJ, Aron DC, Keith RE, Kirsh SR, Alexander JA, Lowery JC. Fostering implementation of health services research findings into practice: a consolidated framework for advancing implementation science. Implement Sci (2009) 4:50. doi: 10.1186/1748-5908-4-50.	6
**81**	Institute for Healthcare Improvement (IHI). SBAR Toolkit. Cambridge, MA: IHI (2018). https://www.ihi.org/resources/tools/sbar-tool-situation-background-assessment-recommendation	7
**82**	Taylor MJ, McNicholas C, Nicolay C, Darzi A, Bell D, Reed JE. Systematic review of the application of the plan-do-study-act method to improve quality in healthcare. BMJ Qual Saf (2014) 23:290–8. doi: 10.1136/bmjqs-2013-001862.	7
**83**	Health Level Seven International (HL7). FHIR overview: Enabling interoperability in healthcare (2024). https://www.hl7.org/fhir/ [Accessed May 15, 2025].	7

**84**	Stacey D, Légaré F, Lewis K, Barry MJ, Bennett CL, Eden KB, et al. Decision aids for people facing health treatment or screening decisions. Cochrane Database Syst Rev. (2017) 4:CD001431. doi: 10.1002/14651858.CD001431.pub5.	2
**85**	American Psychiatric Association. *Cultural Formulation Interview*. 2013. Available from: https://www.psychiatry.org/File%20Library/Psychiatrists/Practice/DSM/APA_DSM5_Cultural-Formulation-Interview.pdf	3
**86**	Flores G. The impact of medical interpreter services on the quality of health care: a systematic review. Med Care Res Rev (2005) 62:255–99. doi: 10.1177/1077558705275416.	7
**87**	Shojania KG, Duncan BW, McDonald KM, Wachter RM, Markowitz AJ. Making health care safer: a critical analysis of patient safety practices. Evid Rep Technol Assess (Summ) (2001) 43:1–668.	1, 7
**88**	Curran GM, Bauer M, Mittman B, Pyne JM, Stetler C. Effectiveness-implementation hybrid designs: combining elements of clinical effectiveness and implementation research to enhance public health impact. Med Care (2012) 50:217–26. doi: 10.1097/MLR.0b013e3182408812.	6
